# Sculpting with stem cells: how models of embryo development take shape

**DOI:** 10.1242/dev.192914

**Published:** 2021-12-15

**Authors:** Jesse V. Veenvliet, Pierre-François Lenne, David A. Turner, Iftach Nachman, Vikas Trivedi

**Affiliations:** 1 Stembryogenesis Lab, Max Planck Institute of Molecular Cell Biology and Genetics, Pfotenhauerstrasse 108, 01307 Dresden, Germany; 2 Department of Developmental Genetics, Max Planck Institute for Molecular Genetics, Ihnestrasse 63-73, 14195 Berlin, Germany; 3 Cluster of Excellence Physics of Life, Technische Universität Dresden, 01307 Dresden, Germany; 4 Aix Marseille University, CNRS, IBDM, Turing Center for Living Systems, 13288, Marseille, France; 5 Institute of Life Course and Medical Sciences, William Henry Duncan Building, University of Liverpool, Liverpool, L7 8TX, UK; 6 School of Neurobiology, Biochemistry and Biophysics, Tel Aviv University, 6997801, Tel Aviv, Israel; 7 European Molecular Biology Laboratories (EMBL), Barcelona, 08003, Spain; 8 EMBL Heidelberg, Developmental Biology Unit, 69117, Heidelberg, Germany

**Keywords:** Morphogenesis, Mechanobiology, Self-organisation, Embryogenesis, Organoids, Somitogenesis, Neural tube, Stem cells, Gastruloids, Stembryogenesis

## Abstract

During embryogenesis, organisms acquire their shape given boundary conditions that impose geometrical, mechanical and biochemical constraints. A detailed integrative understanding how these morphogenetic information modules pattern and shape the mammalian embryo is still lacking, mostly owing to the inaccessibility of the embryo *in vivo* for direct observation and manipulation. These impediments are circumvented by the developmental engineering of embryo-like structures (stembryos) from pluripotent stem cells that are easy to access, track, manipulate and scale. Here, we explain how unlocking distinct levels of embryo-like architecture through controlled modulations of the cellular environment enables the identification of minimal sets of mechanical and biochemical inputs necessary to pattern and shape the mammalian embryo. We detail how this can be complemented with precise measurements and manipulations of tissue biochemistry, mechanics and geometry across spatial and temporal scales to provide insights into the mechanochemical feedback loops governing embryo morphogenesis. Finally, we discuss how, even in the absence of active manipulations, stembryos display intrinsic phenotypic variability that can be leveraged to define the constraints that ensure reproducible morphogenesis *in vivo*.

## Abstract (precis)

**Summary:** *In vitro* engineering of embryo-like structures (stembryos) from pluripotent stem cells offers unique possibilities to understand how boundary conditions that impose geometrical, mechanical and biochemical constraints pattern and shape the mammalian embryo.

## Introduction

Embryogenesis encompasses a complex choreography of lineage decisions and morphogenetic events that need to be tightly coordinated in space and time to give rise to a fully formed foetus. The establishment of embryonic architecture relies on programmed induction and self-organised propagation, with organisms acquiring their shape given boundary conditions that impose geometrical, mechanical and biochemical constraints (Collinet and Lecuit, 2021). Form, forces and fate are dynamically coupled in space and time; mechanically and biochemically induced changes in geometry can feedback into cell fate decisions, thereby (re)shaping transcriptional, signalling and mechanical landscapes as the embryo is (re)sculpted (Busby and Steventon, 2021; Chan et al., 2017; Gilmour et al., 2017; Hannezo and Heisenberg, 2019; Liu and Warmflash, 2021; Sonnen and Aulehla, 2014). Such cross-talk between the local (cell) and global (tissue) scales can have dramatic effects on patterning and global shape.

Dissecting such feedback driving tissue morphogenesis [from the Greek *morphi* (shape) and *gennisi* (emergence)]; the processes that generate tissue organisation and shape) is non-trivial. This is particularly true for mammalian embryos owing to the limited accessibility of the post-implantation embryo *in vivo*, the inherently complex microenvironment, small sample sizes and, in case of human embryos, ethical limitations (Pera, 2017; Shahbazi and Zernicka-Goetz, 2018; Shahbazi et al., 2019). These impediments can be circumvented by constructing embryo-like structures with pluripotent stem cells (PSCs) *in vitro* (reviewed by Baillie-Benson et al., 2020; Ghimire et al., 2021; Shahbazi et al., 2019; Veenvliet and Herrmann, 2021) (Table 1). Several umbrella terms have been suggested for the resulting structures, but consensus has not been reached (Matthews et al., 2021). Here, we refer to the structures as stembryos and the research field as stembryogenesis (Box 1). In contrast to their *in vivo* counterpart, stembryos are easy to access, track, manipulate and scale. The physical, genetic and optical accessibility and statistical power of stembryos facilitates systematic testing of the mechanical and biochemical cues typically thought to be active within their *in vivo* counterpart, and identification of the minimal set of inputs necessary to pattern and shape an embryo.

**Box 1. Stembryogenesis**

We propose ‘stembryo’ (a portmanteau of ‘stem’ cells and ‘embryo’) as an umbrella term for *in vitro* models of embryo development, and refer to the process by which they form as ‘stembryogenesis’. Although we acknowledge that naming should be a community effort, we believe there are good arguments to adopt stembryogenesis as a common denominator. First, by putting ‘stem’ up front it could shift the focus from the embryo to the stem cells, thereby better positioning it as a model system on its own; complementary to, but not replacing the embryo (in contrast to the popular term ‘embryonic organoid’ or ‘embryoid’). Second, it clarifies that it is a new field with its own advantages (and disadvantages), which is not about copying embryos, but merely utilises developmental engineering to gain insights that are difficult or impossible to achieve using traditional embryo research (e.g. the disconnect between genetic programmes and embryo morphogenesis). Third, the term can be extrapolated to research fields (e.g. ‘experimental stembryology’) clarifying the distinction between *in vivo* and *in vitro* research. The latter argument is particularly important because, as the field continues to evolve, we may discover that not all molecular, cellular and morphogenetic processes in stembryos are similar to the embryo, even though the final morphological outcome is embryo-like. We could, for example, be confronted with cases of convergent morphogenesis (reaching the same result through different routes). Alternatively, cells might adapt to the constraints they are facing, which may not necessarily reflect the *in vivo* situation, and/or hijack developmental programmes (e.g. establishment of the germ layers and body plan without *in vivo*-like gastrulation movements). As the field evolves, we will need a term to clearly distinguish synthetic approaches from *in vivo* counterparts while still making clear that the embryo and its models are linked.

**Table 1.**
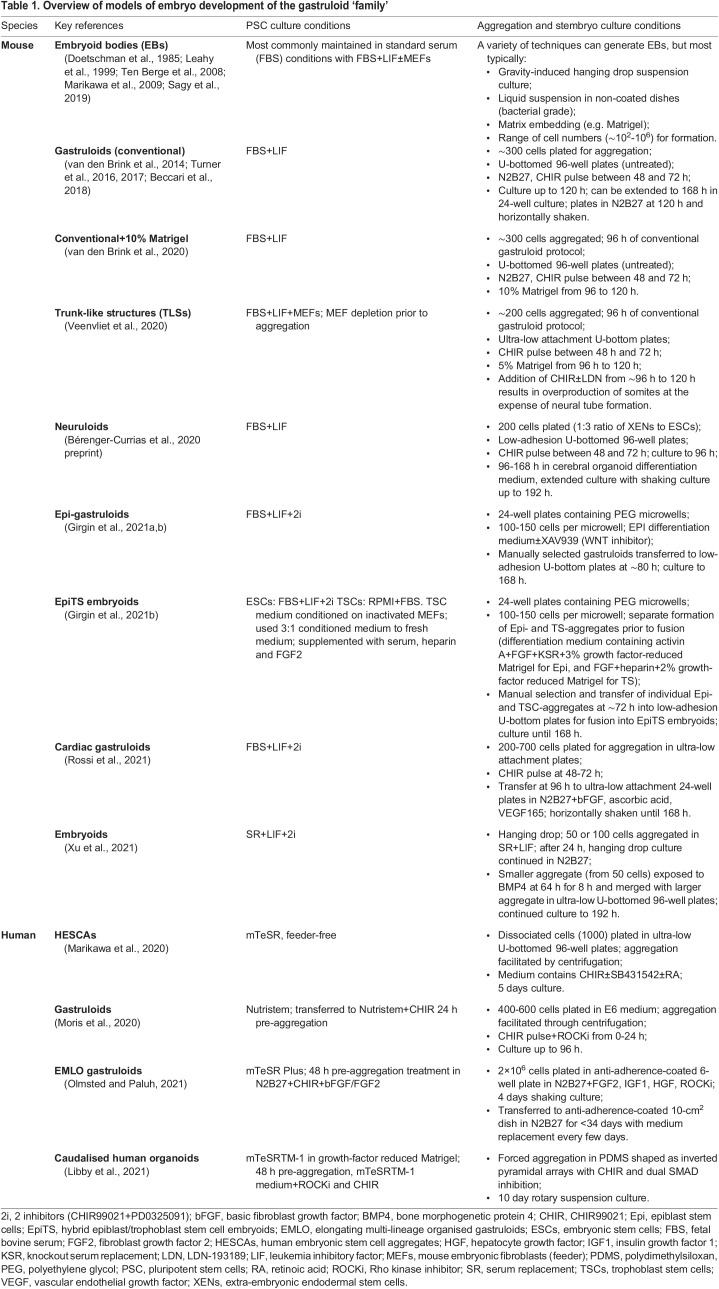
Overview of models of embryo development of the gastruloid ‘family’

In this Review, we detail how combining stembryogenesis with state-of-the-art imaging, genomics, bioengineering devices, biophysical techniques and theoretical modelling can provide an integrative understanding of how the mammalian embryo is reproducibly and correctly sculpted. We focus on stembryos of the gastruloid ‘family’ and their ‘progeny’, which recapitulate the post-implantation stages of embryo development; these are the most challenging to probe *in vivo* because the growth of implanted embryos *in utero* precludes direct observation and manipulation and growth *ex utero* is technically challenging. After a brief description of these models, we first discuss how embryo-like patterning could be achieved. We then explain how modulations of the cellular environment – possibly in balance with cell-intrinsic factors – have resulted in stembryos with distinct levels of embryo-like morphological complexity. We argue that this modularity, combined with the ability to modulate biochemistry, mechanics and geometry actively and precisely at local and global scales, positions stembryos as a unique experimental platform from which to explore and dissect the feedback loops at the heart of embryo morphogenesis. We discuss the experimental and theoretical frameworks necessary to first produce catalogues of fates, forces and flows and then move from correlation to causation by perturbing and controlling this playing field at all of its levels. Finally, we discuss how stembryogenesis permits us to generate a wide variety of tissue organisations and shapes, some of which are not possible in the constrained environment of the embryo. We detail how this effective increase of the accessible phenotypes in morphospace could be leveraged to explore the morphogenetic potential of PSCs and define the physical and genetic constraints that limit this potential *in vivo*.

## Gastruloids: bringing order into embryoid bodies

### From embryoid bodies to gastruloids

Early 3D models of embryo development, termed embryoid bodies (EBs), were formed by aggregation of PSCs (Doetschman et al., 1985; Itskovitz-Eldor et al., 2000; Sánchez et al., 1991). These free-floating embryonic stem cell (ESC) aggregates can differentiate into (derivatives of) the different germ layers, occasionally accompanied by symmetry breaking and the development of an antero-posterior (AP) axis (Boxman et al., 2016; Leahy et al., 1999; Sagy et al., 2019; Ten Berge et al., 2008). However, reproducible induction of such embryo-like events required a further advancement of the EB protocol, resulting in the establishment of gastruloids (van den Brink et al., 2014). Gastruloids trace their origin back to a study in which ensembles of small numbers of P19 embryonic carcinoma cells displayed axial elongation and polarised gene expression (Marikawa et al., 2009). They are formed from similar-sized ESC aggregates and consistent induction of the hallmarks of post-implantation development is ensured by a short pulse (24 h) of the GSK3β inhibitor CHIR99021 (hereafter CHIR; mimicking the downstream consequences of constitutively active WNT signalling) after 48 h of culture (Turner et al., 2017; van den Brink et al., 2014) (Table 1). Gastruloids reproducibly exhibit remarkable self-organising properties, culminating in the formation of the three germ layer derivatives, the establishment of three orthogonal body axes and embryo-like Hox gene collinearity (Beccari et al., 2018; Turner et al., 2017; van den Brink et al., 2014).

Modifications of the mouse gastruloid protocol have permitted the formation of human gastruloids from hESCs, which display axial elongation and generate an AP axis in the absence of extra-embryonic tissues (Moris et al., 2020). Moreover, two separate groups directed their efforts to understanding the development of the nervous system through gastruloid-like approaches. First, combined forced aggregation and rotary suspension culture produces caudalised human organoids recapitulating many of the characteristics of human neural tube development (Libby et al., 2021). Second, by using a shaking culture protocol, elongating multi-lineage-organised gastruloids have been generated, which developed structures with trunk identity, including integrated central and peripheral nervous system correlates (Olmsted and Paluh, 2021) (Table 1).

### How do gastruloids self-organise expression domains?

As highlighted in the last section, both gastruloids and EBs display remarkable self-organisation resulting in spatially confined gene expression patterns. Particularly for the mesodermal marker brachyury (T), both systems converge on a similar pattern despite differences in boundary conditions. For example, in the case of EBs, T expression starts as a polarised pattern in the absence of exogenous WNT activation (through a CHIR pulse). Surface contact biases the location of this T pole in EBs (Sagy et al., 2019). In contrast, in gastruloids (no serum, CHIR pulse), T expression is initiated almost uniformly, and later polarises (Anlas et al., 2021 preprint; Turner et al., 2017).

The resulting T pattern in the two systems is similar, despite arriving through different developmental trajectories, demonstrating the self-organising properties of these systems. This raises the possibility that under distinct mechanochemical conditions, the system uses different mechanisms to converge on a similar pattern. For example, in EBs a local increase near surfaces in the effective concentration of WNT ligand emitted from the cells, as a result of limited diffusion, could jumpstart the WNT-T positive feedback-based loop. In cases in which a pole emerges from an initial ubiquitous T expression pattern (triggered by the CHIR pulse), both reaction-diffusion models (Turing, 1952), as well as wave-pinning models (Ishihara and Tanaka, 2018; Jilkine et al., 2007; Mori et al., 2008) represent attractive possibilities to explain the observed symmetry breaking. These models can describe how fluctuations in cell states in the starting (homogeneous) cell population can lead to the establishment of a pattern within the cell ensemble (Schauer and Heisenberg, 2021).

Another class of mechanisms for spatial segregation of germ layers following spontaneous differentiation (manifested in localised expression domains of corresponding gene markers), is an unmixing mechanism based on differences in surface properties of cells (Foty and Steinberg, 2005; Steinberg, 1970). Thus, it has been proposed that regulated cell adhesion is a driving force for morphogenesis during gastrulation (Hammerschmidt and Wedlich, 2008) and germ layer segregation (Klopper et al., 2010; Krieg et al., 2008; Townes and Holtfreter, 1955). Recent work has demonstrated that such behaviour is conserved in dissociation-reaggregation experiments on hESC-derived 2D gastruloids, micropatterned cultures in which differentiated cells are organised into concentric rings representing the germ layers (Minn et al., 2020). Furthermore, in the case of endoderm formation, islands of E-cadherin (cadherin 1)-expressing cells polarise toward the aggregate tip (gastruloids) or self-sort into clusters or lumens (EBs) through a sorting process (Hashmi et al., 2020 preprint; Pour et al., 2019 preprint). These *in vitro* behaviours capture aspects of embryonic endoderm progression, and its segregation from mesodermal populations.

Deciphering the relative contributions of biochemical and physical mechanisms requires careful measurements, rigorous scrutinisation of underlying assumptions, and appreciation of the differences between *in vitro* and *in vivo* settings. For instance, an unmixing mechanism assumes a fixed cellular identity and ignores possible cross-talk between tissue mechanics and signalling. Furthermore, it has been shown in *Xenopus* that whereas cadherin-based adhesion differences promote cell sorting *in vitro*, dosage compensation of cadherin protein at cell contacts *in vivo* makes differential adhesion insufficient to drive morphogenesis in the embryo (Ninomiya et al., 2012). Other relevant parameters to consider are proliferation and cell shape changes that accompany mammalian gastrulation (Wolpert et al., 1998; Solnica-Krezel and Sepich, 2012): these factors impinge on the spatial pattern and affect local mechanical properties, as seen in the case of mitotic cell-rounding-mediated tissue fluidisation during zebrafish gastrulation, for instance (Petridou et al., 2019).

### Size matters?

**Fig. 1.**
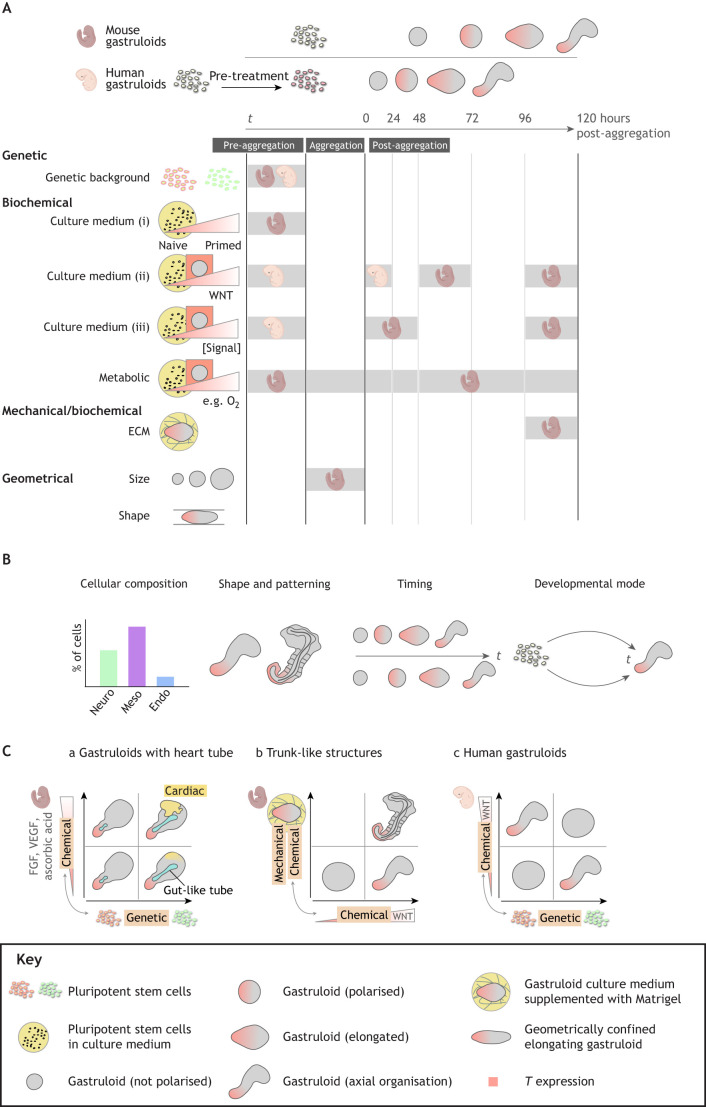
**Cell-intrinsic and -extrinsic determinants pattern and shape the stembryo.** Pluripotent stem cells (PSCs) can be coaxed to form embryonic organoids with distinct levels of morphogenetic complexity, which we term stembryos. (A,B) The cellular complexity, patterning and shape of the stembryo at the culture endpoint (outcome), as well as the route (timing, mode) towards this outcome (process) (B) is the result of a complex interplay of cell-intrinsic and -extrinsic determinants that can be genetic (e.g. PSC genetic background), biochemical (e.g. pluripotent culture conditions, stembryo culture medium and conditions, endogenous secreted and exogenous added signals, ECM composition), mechanical (e.g. ECM stiffness, material properties) and geometrical (e.g. aggregate size, exogenous shape constraints) in nature (A). The grey bars depict the (timing of) modulations experimentally proven to impact culture outcome in mouse and human stembryo systems. Evidence available in the literature for mouse and human, as indicated. (C) Cell-intrinsic and -extrinsic determinants interact. For instance, in cardiac gastruloids (Ca), the induction of heart tube from cardiac tissue (yellow) through addition of cardiogenic factors (chemical modulation) happens in the context of physiological interactions with the gut-like tube (blue) (Rossi et al., 2021). Because the propensity to induce both gut and cardiac cell states in gastruloids is linked to mESC genetic background (e.g. van den Brink et al., 2020), it is conceivable that genetic and biochemical inputs interact in the sculpting of the heart tube. The induction of trunk-like-structures (Cb) requires the interaction of chemical (activation of WNT signalling through CHIR pulse), as well as mechanochemical [addition of ECM components (Matrigel)] constraints (Veenvliet et al., 2020). Finally, in human gastruloid formation (Cc), distinct hESC lines require different levels of WNT activation (through CHIR), both pre-aggregation and post-aggregation, pointing to an interdependence of cell-intrinsic genetic and extrinsic chemical determinants (Moris et al., 2020). Note that for all examples shown, geometrical constraints, in the form of controlled aggregate size, are also necessary. Endo, endoderm; Meso, mesoderm; Neuro, neuro-ectoderm. Mouse and human embryo schematics from BioRender*.*

A crucial difference between gastruloids and previous EB work (e.g. Ten Berge et al., 2008), was the number of cells used, with lower amounts (200-300 cells) used per aggregate than traditional EB culture (Fig. 1A). This number is similar to the number of epiblast cells (150-300) in the embryonic day 5.5 mouse embryo (Davidson et al., 2015; Muñoz-Descalzo et al., 2015). This permitted the signalling conditions required for robust symmetry breaking, polarised gene expression (specifically of posterior markers such as *T* and *Wnt*), and subsequent axial elongation (Marikawa et al., 2009; van den Brink et al., 2014). Nonetheless, recent work suggests that, at least within certain limits (50-1000 cells), T expression is not regulated by size-dependent signal gradients (Boxman et al., 2016; Anlaş et al., 2021 preprint). In contrast, EB size is an important parameter in governing the endothelial versus cardiac lineage decision via differential expression of non-canonical WNT pathway members (Hwang et al., 2009). Similarly, *de novo* pattern formation due to cadherin-mediated cell sorting is size dependent: only smaller aggregates (1000 cells, similar order of magnitude as gastruloids) obtain two-domain patterns, whereas larger aggregates (10,000 cells) result in complex patched patterns (Cachat et al., 2016; Davies, 2017). Thus, their modulatory nature renders stembryos useful for studying how system size impacts self-organising mechanisms, for defining the lower and upper boundaries allowing pattern formation, and for understanding how these might be distinct for different cell and tissue types.

In summary, the spatial segregation of germ layers in stembryos can be achieved through multiple mechanisms, which might be system specific. Careful dissection of the biophysical and biochemical inputs guiding this segregation, as well as the system's scaling capacities, will shed light on the processes that dictate the emergence of organised gene expression domains *in vitro*, and provide entry points for assessing the relevant parameters *in vivo*.

## Changing the cellular environment coaxes stembryos into shape

Although gastruloids mimic the post-occipital axial development of the post-implantation mouse embryo, both temporally and spatially, initial structures lacked typical aspects of embryo architecture. For instance, in conventional gastruloids, cardiac mesoderm does not form a heart tube (Rossi et al., 2021), pre-somitic mesoderm does not condense into somites (Beccari et al., 2018), and neural cells do not organise into a neural tube (Beccari et al., 2018). This apparent uncoupling of genetic programmes and tissue morphogenesis suggests that crucial inputs driving embryo architecture are missing in conventional gastruloids. Indeed, recent work has demonstrated how the morphogenetic potential of gastruloids is unlocked by changing the gastruloid formation conditions, in particular the cellular environment (Table 1; Fig. 1A,B).

### Chemical modulation

Multiple aspects of embryo-like architecture in stembryos have been achieved by altering the culture medium. Anterior neural tissues, absent in conventional gastruloids, can be formed through WNT inhibition during early gastruloid development (Girgin et al., 2021a). It should be noted that WNT inhibition is not the only aspect that distinguishes the generation of these ‘epi-gastruloids’ from conventional gastruloids: aggregates are formed under different media conditions (activin A, FGF2, KSR) in hydrogel microwell arrays (Girgin et al., 2021a). The same group demonstrated how addition of cardiogenic factors resulted in the formation of a heart tube. This morphogenetic event occurs in the context of physiological multi-tissue interactions, in particular the association between the cardiac crescent and a putative primitive gut-like tube (Rossi et al., 2021). The formation of the primitive gut-like tube itself appears to be a self-organising process in both mouse and human stembryos (Hashmi et al., 2020 preprint; Vianello and Lutolf, 2020 preprint; Olmsted and Paluh, 2021). Interestingly, the efficiency of formation of a heart tube and gut-like structure might depend on the interplay between cell-intrinsic and -extrinsic factors (discussed in more detail below; Fig. 1Ca).

### Co-culture approaches

Co-assembly of distinct stem cell types or differentially treated ESC aggregates can also increase the cellular and/or morphological complexity of stembryos. Fusing naive ESC aggregates with a slightly smaller BMP4-treated ESC aggregate functioning as a morphogen signalling centre resulted in gastruloid-like stembryos including a notochord and cephalic structures (both absent in conventional gastruloids; Xu et al., 2021). Co-assembly of ESCs with extra-embryonic endoderm stem cells induces the formation of anterior neural tube-like structures that can further differentiate into cerebral cortex-like tissue when cultured in appropriate media (Bérenger-Currias et al., 2020 preprint). Notably, other co-assembly approaches have included trophoblast stem cells (blastoids: Rivron et al., 2018; ETS/ETX/iETX stembryos: Harrison et al., 2017; Sozen et al., 2018; Amadei et al., 2021). Interestingly, these models do more reliably mimic the architecture of the pre- and peri-implantation embryo, but (at present) cannot further develop into the later developmental stages modelled by gastruloids. This might be due to constrained development in the co-culture setting, which is absent in the culture of stembryos of the gastruloid ‘family’ (Anlas and Trivedi, 2021).

### Providing extracellular matrix components

Complex embryo-like architecture can be achieved by adding Matrigel, an extracellular matrix (ECM) of natural origin with basement-membrane proteins laminin, collagen IV, entactin and the heparin sulphate proteoglycan perlecan as major constituents (Aisenbrey and Murphy, 2020). *In vivo*, the ECM provides biochemical and mechanical cues that regulate the morphological properties of cells and tissues (reviewed by Walma and Yamada, 2020). *In vitro*, Matrigel can substitute for the ECM inputs normally present in the tissue's natural environment, resulting in complex morphogenesis in organoids (Fig. 1A,B) (Brassard and Lutolf, 2019; Eiraku et al., 2011; Kleinman and Martin, 2005; Meinhardt et al., 2014). In gastruloids, precisely timed addition of 5% Matrigel to the culture medium induces embryo-like architecture with somite-like structures that form as epithelial spheres comprising apico-basal polarised cells, juxtaposed to a neural tube-like structure. These have been termed trunk-like-structures (TLSs) for their resemblance to the embryonic trunk (Fig. 1Cb) (Veenvliet et al., 2020). Parallel independent work has demonstrated that adding 10% Matrigel to the culture medium transforms the organised spatial expression domains of gastruloids into a single band of somite-like structures organised as a series of discs along the AP axis (van den Brink et al., 2020). The distinct architectures achieved in both culture systems could be indicative of a ‘sweet spot’ for the mechanochemical constraints imposed by Matrigel addition, which is further supported by titration experiments that demonstrate reduced efficiency of somite formation if the Matrigel percentage is increased (van den Brink et al., 2020). Additional evidence for the importance of precise tuning of the mechanochemical properties of the matrix comes from recent work demonstrating that compliant substrates promotes the self-organisation of human ESCs into gastrulation-like nodes with cellular behaviours highly reminiscent of *in vivo* gastrulation (Muncie et al., 2020). However, a (dominant) role for other cell-intrinsic and -extrinsic determinants in dictating the distinct architectures should not be excluded.

### Cell-intrinsic and -extrinsic determinants orchestrate cellular and morphological complexity

Although our knowledge of stembryo formation is limited, current evidence points to a close association of cell-intrinsic and -extrinsic determinants in orchestrating stembryo morphogenesis. The TLS protocol not only uses a different percentage of Matrigel, but also genetically different ESCs cultured under distinct pluripotency conditions (van den Brink et al., 2020; Veenvliet et al., 2020; Table 1). A further example for cell-intrinsic determinants is the formation of gut primordia in stembryos, which is likely correlated with the ESC genetic background (discussed by Veenvliet and Herrmann, 2021). Although for stembryos this evidence is anecdotal, observations from 2D directed differentiation assays support the idea of the ESC genetic background as a driver of distinct differentiation capacities under identical culture conditions (Ortmann et al., 2020). This phenotypic variability can be explained partially by inconsistent activity of extracellular signalling, such as the WNT pathway. Interestingly, different human ESC lines require treatment with distinct concentrations of CHIR for efficient human gastruloid formation, both pre- and post-aggregation (Moris et al., 2020), suggesting that modulation of WNT signalling partially cancels out the effects of the PSC genetic background (Fig. 1Cc). Further evidence for interaction of cell-intrinsic and -extrinsic determinants comes from recent work showing that treatment of gastruloids with FGF2 during ESC aggregation results in robust induction of FOXA2^+^ tubular structures, reminiscent of gut tubes (Gharibi et al., 2020 preprint). Similarly, culturing of gastruloids under hypoxic conditions vastly increased the efficiency of gut-tube-like structure formation (López-Anguita et al., 2021 preprint). Thus, stembryos can be exploited to test how cell-intrinsic distinct differentiation capacities can be tamed by modulating the cellular environment, possibly providing insight into the constraints that ensure robust ratios of the three germ layers *in vivo* (Fig. 1A-C).

## Connecting fates, forces and flows: bridging local and global scales in stembryos

**Fig. 2.**
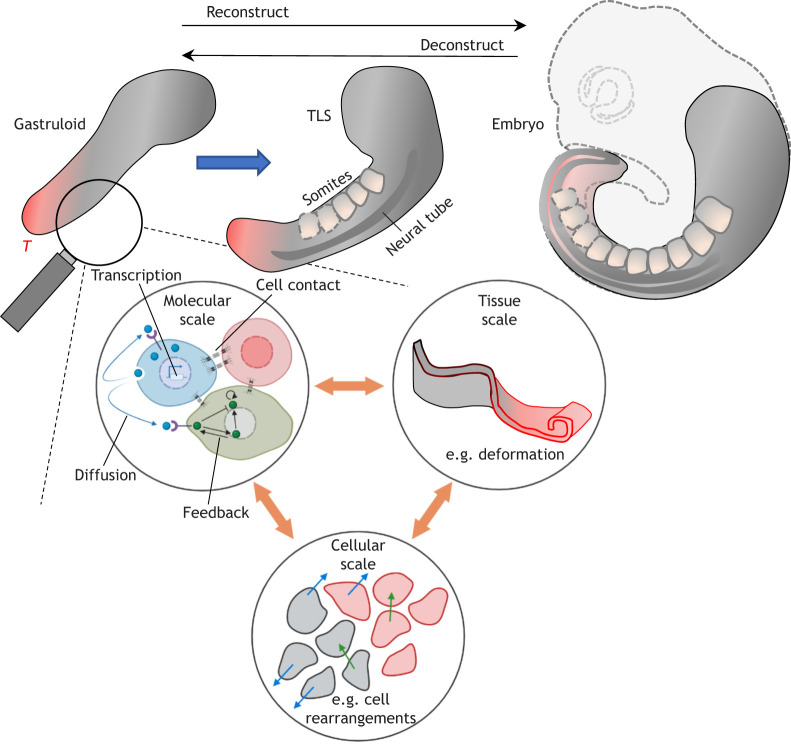
**Bridging local and global scales in stembryos.** Stembryos allow us to probe processes at multiple scales (molecular, cellular and tissue-level) to disentangle the genetic, mechanical, biochemical and geometrical inputs that shape the (st)embryo. At the molecular level, processes such as transcription, protein synthesis, localisation, secretion, molecular diffusion, inter- and intracellular interactions and regulatory networks define the molecular state of the cells. Cellular behaviour in terms of movement, rearrangements and mechanical coupling between cells that dictate neighbour interactions translate into macroscopic-tissue level properties that are essential for deformations and movements that shape the tissue during (st)embryo development. Understanding these multi-scale interactions and feedback mechanisms holds the key to reconstructing and deconstructing the processes that underlie morphogenesis. Schematic of the mouse embryo adapted from Gritti et al. (2021). Parts of the figure generated using BioRender.

**Fig. 3.**
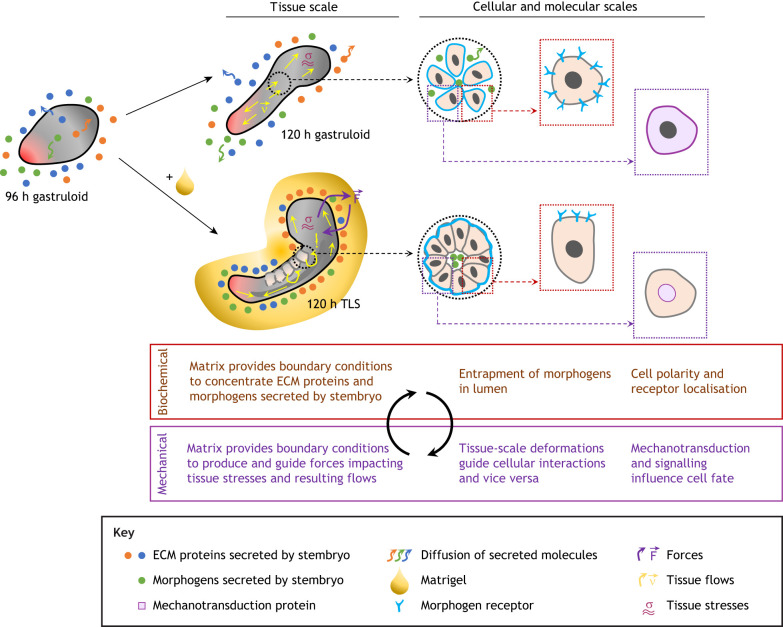
**Comparative mapping of morphogenetic information modules across scales.** Direct comparative analysis of stembryos with distinct degrees of morphological complexity might provide insights into the design principles that convey embryo architecture. For example, as shown for TLS the addition of ECM components (Matrigel) can concentrate morphogens and ECM proteins secreted by the stembryo at the structure-matrix interface (e.g. as observed for fibronectin in TLSs; Veenvliet et al., 2020). In conventional gastruloids, these proteins would freely diffuse into the medium in the absence of a physical boundary. Concomitantly, the formed ECM provides boundary conditions to produce and guide forces, impacting tissue stresses and resulting flows that, in turn, lead to deformations that could trigger morphogen release, alter cellular interactions (e.g. by changing the apposition of signalling and responding tissues), and/or feedback into local scales through nucleocytoplasmic shuttling of mechanotransduction proteins, affecting cell fate decisions. In addition, alterations in tissue architecture might spatially constrain morphogen signalling, for example through entrapment of morphogens in formed lumen, or by restriction of receptor localisation concomitant with the establishment of apico-basal polarity of somitic and neural cells. Note that, although only the feedback between the biochemical and mechanical information modules is shown here, at various levels the loop feeds back into the genetic (e.g. mechanotransduction and signalling influencing cell fate) and geometrical (tissue-scale deformations) information modules (see main text and Box 2 for more details).

In multicellular systems without fixed boundaries, including mammalian embryos and stembryos, local changes in cell behaviours, such as cell divisions or cell movements, inevitably deform boundaries, which, in turn, can affect the internal arrangements by producing and guiding forces (Collinet and Lecuit, 2021; Lenne et al., 2021; Trepat and Sahai, 2018). These forces can direct biochemical signalling and cell fate decisions through, for example, mechanotransductive pathways (Chan et al., 2017; Kumar et al., 2017; Vining and Mooney, 2017). Concomitantly, the (de)formation of physical boundaries can reshape the signalling landscape of stembryos by altering the apposition of signalling and responding tissues (Busby and Steventon, 2021; Chan et al., 2017; Kumar et al., 2017). Thus, cross-talk between morphogenetic information at the local (cell) and global (tissue) scales can have dramatic effects on patterning and global shape (Fig. 2; Fig. 3). Whereas the complex microenvironment impedes a detailed understanding of the feedback mechanisms *in vivo*, stembryos offer a unique experimental assay to bridge the different scales and dissect mechanochemical feedback loops governing (st)embryo morphogenesis for two main reasons. First, the possibility to control precisely the biochemical and biophysical properties of the cellular environment, and the accessibility to optical imaging, can be leveraged to map how imposing *in vivo*-like boundary conditions sculpt the stembryo across spatial and temporal scales (Box 2; Fig. 2; Fig. 3). Second, stembryos are amenable to both local and global perturbation, enabling the establishment of causal relationships. In this section, we outline which existing methodology should be implemented and complemented with new experimental and theoretical tools to exploit optimally this huge potential of stembryology (Table 2).

**Box 2. Catalogue of morphogenetic information modules**

Cataloguing differences in the information modules harbouring morphogenetic information (genetics, biochemistry, mechanics, geometry) (Collinet and Lecuit, 2021) in stembryos with distinct levels of morphological complexity can provide insights into the processes that sculpt the (st)embryo (Fig. 3). Methods to map (e.g. single-cell RNA sequencing) and manipulate (e.g. CRISPR-Cas9) the genetics module are well-established. Comparative genomics can link distinct stembryo architecture to transcriptional changes (Bérenger-Currias et al., 2020 preprint; Girgin et al., 2021a,b; Veenvliet et al., 2020). However, such analyses should be complemented by a (comparative) inventory of biochemistry and mechanics (Gorfinkiel and Martinez-Arias, 2021).

**Table 2.**
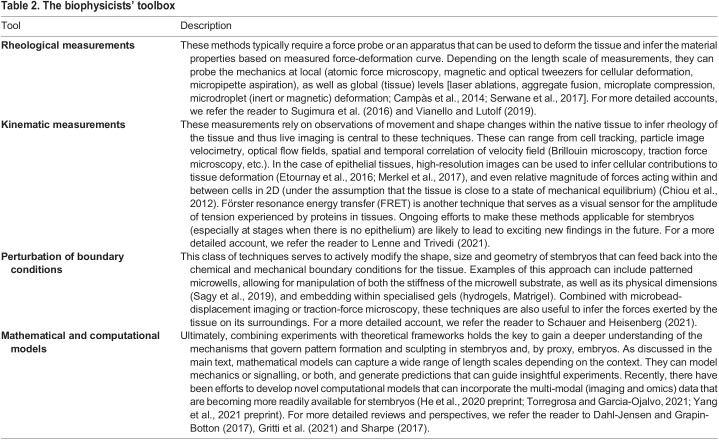
The biophysicists' toolbox

For example, in TLSs addition of ECM alters localisation, but not expression level, of the ECM protein fibronectin (Fig. 3) (Girós et al., 2011; Molè et al., 2020; Veenvliet et al., 2020), and in gastruloids the developing gut primordia deposit and organise their own matrix (Vianello and Lutolf, 2020 preprint). Matrix supplementation/deposition might constrain morphogen diffusion by concentrating them in the extracellular space (Fig. 3) (Rozario and DeSimone, 2010; Brizzi et al., 2012). Lumen formation could entrap diffusible proteins (Durdu et al., 2014; Shyer et al., 2015). Finally, induction of cell polarity might impact receptor localisation (Etoc et al., 2016; Zhang et al., 2019; Veenvliet et al., 2020) (Fig. 3). To explore these possibilities, readily accessible methods to map and manipulate biochemical signals, such as biosensors and bathing in small molecule inhibitors, should be complemented with opto- and chemogenetics to achieve better spatial and temporal control (Hartmann et al., 2020; Martinez-Ara et al., 2021 preprint; Mumford et al., 2020; Repina et al., 2019 preprint; Shiri et al., 2019). Implementation of synthetic morphogen systems would provide control of different morphogen parameters (Manfrin et al., 2019; Stapornwongkul and Vincent, 2021; Stapornwongkul et al., 2020; Toda et al., 2020; Zheng et al., 2019).

Architectural changes associated with manipulations of the cellular environment likely impact flows, forces and force transduction (Fig. 3). Theoretical work has attempted to understand shape changes in ECM-embedded spheroids as a result of competition between interfacial tension and forces exerted by the matrix (Parker et al., 2020 preprint). Such physical boundaries and timely changes in their mechanical properties contributes to shaping and patterning of the elongating axis *in vivo* (avian: Kunz et al., 2021 preprint; Xiong et al., 2020; mouse: Hiramatsu et al., 2013; Kyprianou et al., 2020; Matsuo and Hiramatsu, 2017; zebrafish: Mongera et al., 2018; Thomson et al., 2021). Importantly, supra-cellular forces not only alter tissue shape, but also drive cell shape changes, which in turn feed back onto tissue shape (Sanematsu et al., 2021).

### Experimental need

#### Mapping stress patterns

A combined understanding of the stresses in tissues and the material properties that dictate the response to such stresses can help us understand the mechanical basis of shape in stembryos. Methods for stress measurements in living tissues are now mature enough to map stress patterns in the stembryo (Campàs, 2016; Gómez-González et al., 2020; Sugimura et al., 2016). Forces at different length scales can be measured by laser ablation assays, with the direction and velocity of recoil providing information on the forces acting prior to ablation (Grill et al., 2001). However, this method is destructive and therefore not suited for mapping physical forces over time. An alternative is provided by incorporating deformable microspheres, either soft polymeric beads (Mohagheghian et al., 2018) or magnetic oil droplets (Serwane et al., 2017), into tissues, a technique that has been successfully used to map the stresses that act at cellular and supra-cellular scales during zebrafish axial elongation (Kim et al., 2021; Mongera et al., 2018; Shelton et al., 2021 preprint). In many cases, understanding the response of tissues to stresses demands the knowledge of the material properties of the tissue, which can be inferred by measuring strain of the tissue under a known stress. Several techniques could be implemented for such measurements in stembryos, ranging from cellular to supra-cellular levels, such as optical manipulation (Bambardekar et al., 2015), magnetic actuation of droplets (Serwane et al., 2017; Mongera et al., 2018), micropipette aspirations (Guevorkian et al., 2010), tissue coalescence (Jakab et al., 2008; Oriola et al., 2020 preprint), parallel plate compression (Forgacs et al., 1998) and axisymmetric drop analysis (David et al., 2009).

#### Mapping cellular movements and tissue flows

Complementary to direct measurement of the forces, cellular movements and tissue flows can be reconstructed by tracking cells labelled with ubiquitous nuclear or membrane reporters in stembryos live imaged *in toto* by multi-photon or light-sheet microscopy (Anlaş et al., 2021 preprint; de Medeiros et al., 2021 preprint; Hashmi et al., 2020 preprint; He et al., 2020 preprint; McDole et al., 2018; Samal et al., 2020; Serra et al., 2019; Shah et al., 2019). Whereas global tissue flows can be measured using particle image velocimetry or optic flow (Hashmi et al., 2020 preprint), monitoring tissue-level movements at single-cell resolution is still more challenging (de Medeiros et al., 2021 preprint; McDole et al., 2018; Shah et al., 2019). Here, the ease of generating mosaic stembryos with a fully controllable percentage of reporter-expressing cells will be useful to facilitate reliable tracking of single cells. Importantly, live-imaging data of non-muscle myosin can be employed to relate morphogenetic flow to the patterns of force generation (Behrndt et al., 2012; Münster et al., 2019; Streichan et al., 2018). However, the importance of combining flow inference with experimental measurement of the flow field was recently demonstrated in the *Tribolium* embryo, in which a mismatch between the two could be resolved by predicting a previously overlooked fixed boundary, which was experimentally confirmed (Münster et al., 2019).

#### Defined modular matrices to disentangle biochemical and biophysical inputs

Currently, induction of embryo-like architecture in stembryos relies on the use of Matrigel; thus, biochemical and mechanical cues are inevitably coupled (van den Brink et al., 2020; Veenvliet et al., 2020). For instance, increasing matrix stiffness by using a higher percentage of Matrigel also increases the concentration of growth factors that are components of Matrigel (Hughes et al., 2010; Vukicevic et al., 1992). Thus, to disentangle the mechanical and chemical inputs, the use of 3D synthetic modular matrices, such as polymeric hydrogels supplemented with chemical components, is required to enable the tuning of biochemical (e.g. morphogen gradients) and biophysical (e.g. stiffness) parameters separately (Ashworth et al., 2020; Brassard and Lutolf, 2019; Gjorevski et al., 2016; Ranga et al., 2016; Simunovic et al., 2019). In addition, artificial sources of localised signals could be employed to disentangle the contributions of mechanical and biochemical inputs through spatial separation of the two sources. For example, a patch of Wnt3a- or Dkk1-emitting cells can bias the location of the T induction domain, which is otherwise determined by the contact point of the EB with its surroundings, towards or away from the cell patch, respectively (Sagy et al., 2019).

#### Active perturbation of architecture

As one of the most striking differences between embedded and non-embedded gastruloids is their distinct architecture, direct comparative analysis might unveil the impact of embryo-like geometry on cell fate specification and behaviour (Fig. 3; Box 2). Although this method is excellently suited to index changes in forces, fate and form, it may prove challenging to dissect cause and consequence. To this end, it is essential to actively perturb the feedback loops between the morphogenetic information modules governing (st)embryo architecture, by actively changing geometry, for instance. This method has been successfully applied to link form to forces and fate in 2D micro-patterned cultures and mouse blastomeres (Blin et al., 2018; Lenne et al., 2021; Muncie et al., 2020; Royer et al., 2020). Combining geometric perturbation and measurement of cell movements with genetically encoded fluorescent biosensors should, for example, enable dissection of how geometry-guided tissue flows alter the apposition of inducing and responding cells, or how geometric changes are detected by mechanotransductive pathways, to ultimately impact cell fate. Such a direct visualisation of the feedback loop at all of its levels can bridge it from the global to the local scale. Similarly, optogenetics can be employed to perturb biochemical pathways with high spatiotemporal control at the local scale, and study effects globally (e.g. Martinez-Ara et al., 2021 preprint).

### Modelling need

#### Relating mechanical stresses to deformation and flows

Physical models and their computational implementation will be essential in order to relate mechanical stresses to deformation and flows (Dahl-Jensen and Grapin-Botton, 2017; Sharpe, 2017). Several models have been proposed to describe tissue mechanics (for a detailed overview of the use of such models in *in vitro* systems, see Gritti et al., 2021). Agent-based models, either particle-based or vertex models (Buske et al., 2012; Farhadifar et al., 2007; Okuda et al., 2018; Thalheim et al., 2018), aim to explain multicellular (higher length scale) phenomena based on interactions between individual cells (smaller length scale). Vertex models represent a large class of discrete models that consider cells as individual objects and their mechanical interfaces (reviewed by Fletcher et al., 2014). They are particularly suited for relating local cell mechanics to tissue deformation, and are therefore valuable to stembryogenesis. An important recent advance is the ability to account for extracellular spaces, complex cell shapes and tension fluctuations at cell-cell contacts in a fully dynamic vertex model (Kim et al., 2021). This model reproduced many of the cell- and tissue-scale behaviours that are experimentally observed during zebrafish axial elongation, and revealed that tension fluctuations control tissue rigidity phase transitions (Kim et al., 2021; Mongera et al., 2018; Petridou et al., 2021).

Continuum models that consider the cell collectives as a continuum material are likely to be more appropriate for quantitative predictions about system behaviour and its dependence upon changes in size, shape and boundary conditions. Although these models primarily describe tissue-scale cell mechanics, an equally rich body of work describes mechanics at (sub-)cellular levels and they rely on modelling of molecular interactions. For example, clutch models can explain cell movement and durotaxis by accounting for substrate stiffness, adhesion proteins, myosin motors and actin cytoskeleton that form the molecular basis of cell-substrate interactions (Chan and Odde, 2008). At the sub-cellular level, polymer network models describe and predict cytoskeletal properties and nonlinear mechanical responses to compressive and tensile stresses (Belmonte et al., 2017; Gardel et al., 2004; Wollrab et al., 2018).

#### Bridging length scales

In order to understand how local changes in material properties affect global changes and, in turn, feedback onto smaller length scales, bridging length scales is pivotal (reviewed by Trepat and Sahai, 2018). Limited, but promising, attempts to explain tissue deformation as a result of changes in local properties have been made in *Drosophila* through a combination of careful biophysical measurements and genetic perturbations (Clément et al., 2017; Lebreton et al., 2018). On the theoretical side, there have been efforts to connect length scales through coarse-graining approaches (Alt et al., 2017; Hannezo et al., 2014; Murisic et al., 2015). To date, such efforts have been mostly limited to 2D epithelial tissues; it will require concerted efforts of both experimentalists and theorists to account for complex three-dimensional multicellular systems, such as embryos and stembryos, which harbour cells in epithelial, mesenchymal and transitory states, with extracellular spaces that change dynamically.

#### Coupling of different timescales

Another crucial factor to account for is the coupling of different timescales: the timescale for changes in microscopic properties (e.g. cytoskeletal rearrangement, cell movement, expression of cell-surface proteins) can affect the population level behaviour at a different timescale. The dependence on molecular processes to alter intercellular connections within the tissue dictates the timescale for tissue-level changes in terms of solid-like and fluid-like states (Bénazéraf et al., 2010; Bi et al., 2015; Mongera et al., 2018; Petridou et al., 2019; reviewed by Petridou and Heisenberg, 2019; Lenne and Trivedi, 2021). In confluent monolayers, the relative magnitudes of molecular-scale T1 delay [the time a cell needs to execute the molecular processes for neighbour exchanges (T1 transition)] and the cell-scale collective response timescale render the tissue elastic-like or fluid-like and thereby dictate the cellular patterns (Erdemci-Tandogan and Manning, 2021).

#### Incorporating gene regulatory networks in models of tissue mechanics

Finally, incorporating gene regulatory networks in models of tissue mechanics, while still accounting for cell movements, requires strong interactions between experimentalists and theorists. Specialised theoretical tools need to be implemented depending upon the concentration of the relevant molecules that form part of the morphogenetic fields. The concentration of the chemical species (or the number of activities/interactions) can be sufficiently high to justify it as a continuous variable for modelling with differential equations (Gierer and Meinhardt, 1972; Turing, 1952). This approach has been used successfully in many different contexts (Meinhardt, 2008), and can be combined with cell rearrangement data to explain the appearance of gene expression domains (Fulton et al., 2021 preprint). Alternatively, the relatively small absolute number of chemical reactions renders the process noisy and therefore stochastic modelling (Gillespie, 1977) has also been used to model morphogen signalling (Barone et al., 2017).

### Further considerations

Overall, bridging local and global scales in stembryos is an exciting and much-needed research direction that builds on molecular biology, engineering and physics (Gritti et al., 2021; Gupta et al., 2021; Schauer and Heisenberg, 2021; Torregrosa and Garcia-Ojalvo, 2021 preprint). It is, however, important to recognise the limitations and the underlying assumptions of existing techniques. First, most cells in conventional gastruloids do not display clear epithelial organisation, thereby limiting immediate applicability of several theoretical and measurement tools that have been used successfully in, for example, *Drosophila*. Interestingly, such tools may be more readily applicable in TLSs, in which the majority of the cells display clear epithelial organisation (Veenvliet et al., 2020). When developing theoretical/computational models of the stembryos*,* it also becomes essential to consider that the coupling of timescales of changes in material properties and that of deformation (i.e. response to stresses) in living matter can happen quite distinctly from inert materials, in which the mechanical properties are generally constant. Population level rheological properties can be concomitant with changes in the tissue shape, thereby making the predictions about tissue deformation extremely non-trivial owing to the force field generated and experienced by the constituent cells (Lenne and Trivedi, 2018). Finally, transcriptionally similar cells in stembryos and embryos may have different absolute mechanical properties, yet it is conceivable that the relative property differences between such cells and their environment facilitate similar pattern- and shape-forming mechanisms.

## Learning from variation: exploring the stembryo morphospace

**Fig. 4.**
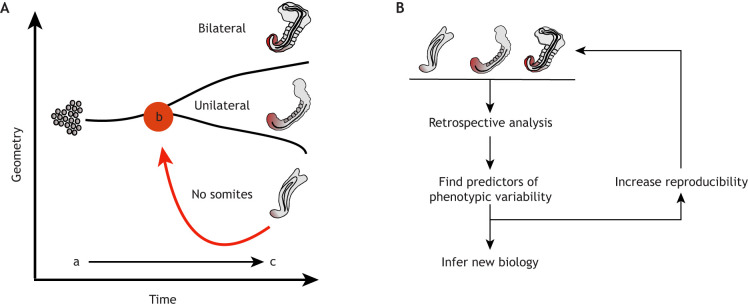
**Exploring the stembryo morphospace.** (A) Stembryos can settle into different molecular and morphological states. For example, TLSs can settle into states with no somites, unilateral somites or bilateral somites at time c. The points at which small changes in the cellular environment have driven these distinct morphological outcomes in phase space can be conceptualised as bifurcation points (time b), which can be identified by backtracking from the attractor states (see main text). (B) Such retrospective analysis can identify the developmental dynamics driving variation, which can be used to: (1) infer new biology by leveraging the variation leading to deviation of the embryo ground truth (for the example shown in A, ‘How is bilaterality reproducibly achieved *in vivo*?’); and (2) make spontaneous variation controllable by identifying targetable sources of variation.

We have illustrated how the absence of *in vivo* constraints allows stembryos to explore a broader spectrum of possible forms, and how controlled addition of *in vivo*-like constraints can result in morphologies more closely resembling (but not copying) the embryo. This collection of possible morphological outcomes can be conceptualised as a ‘morphospace’ (discussed for organoids by Jabaudon and Lancaster, 2018; Ollé-Vila et al., 2016). In the previous sections, we have presented experimental and theoretical frameworks to pinpoint the developmental processes underlying the distinct phenotypic outcomes triggered by controlled variations of the cellular environment (e.g. addition of ECM components) and/or known cell-intrinsic determinants (e.g. ESC genetic background). However, in addition to experimentally induced variation, stembryos, like many organoid systems, display natural heterogeneity resulting in phenotypic variability (i.e. different morphological outcomes despite identical culture conditions). Identifying the causative developmental dynamics of such spontaneous variation is useful, as it may reveal the (epi)genetic and physical constraints that control and limit variability *in vivo*. For example, TLSs generated in the same experiment can develop unilateral, bilateral or no somites (Fig. 4A). Because bilaterality is reproducibly achieved in the embryo, understanding what drives the deviation of this ‘ground truth’ in stembryos can inform us about the processes that ensure bilateral symmetry *in vivo*.

### Identifying the developmental dynamics underlying variation by backtracking bifurcation points

In order to harness phenotypic variability for extracting biological information, two advantages of stembryos need to be combined: statistical power and the possibility of automated real-time analysis. Moreover, tools need to be developed that digitise the data and define the parameters that describe the attractor states in phase space (reviewed by Dahl-Jensen and Grapin-Botton, 2017). In stembryo morphospace, the points at which small changes in the cellular environment change the morphological outcome can be conceptualised as bifurcation points. Identifying these bifurcation points is important in order to track down the causes of phenotypic variability (Fig. 4). We envision that automated arrayed platforms, in combination with computer vision and machine-learning approaches, can be employed to backtrack the bifurcation points from the attractor states in stembryo culture systems (Lukonin et al., 2020). The identification of the molecular, cellular and morphogenetic processes that underlie the bifurcations, and are thus predictive of the attractor states, will provide important insights into the constraints that control the reproducible morphological outcome *in vivo* (Fig. 4B).

### Reaching similar attractor states through distinct developmental modes?

It is important to remember that even in those cases in which the morphological outcome of the process is reminiscent of an embryo (i.e. an *in vivo*-like body plan with an axial neural tube flanked by bilateral rows of somites), the stembryo may not necessarily employ the same developmental mode to reach this state. In contrast, stembryos may engage parallel but distinct modes that, nevertheless, ultimately converge on the same body plan (Anlas and Trivedi, 2021). Employment of different developmental trajectories to reach a similar morphological state has been observed in *Nematostella vectensis* dissociation-reaggregation experiments, whereby aggregates of dissociated gastrula cells use an alternative developmental mode normally reserved for distantly related members of the same phylum (Kirillova et al., 2018). Although the manifold developmental trajectories engaged by stembryos to achieve the same (or similar) body plan have not yet been identified, it is conceivable that the grand sum of developmental modes engaged by stembryos to establish an *in vivo*-like body plan is a mere reflection of the morphogenetic capacities of the cells given the constraints they are faced with (or lack thereof) (Box 1). Defining the stembryo morphospace and understanding how cellular ensembles reach the attractor states can thus teach us important lessons about the developmental plasticity of embryonic cells by unmasking what cells can do once *in vivo* constraints are removed. This has already resulted in important lessons, such as the apparent disconnect between genetic programmes and embryo morphogenesis (Moris et al., 2021; Veenvliet and Herrmann, 2021). To exploit this potential fully, it will be important to develop frameworks that allow for *in toto* parallel recording of cellular behaviour and state.

### Further considerations

The phenotypic landscape of stembryos could be further broadened (or possibly condensed) by experimental perturbations. For instance, activation of WNT during the window of Matrigel addition results in a phenotype otherwise not observed in TLSs, with excess somites arranged as a ‘bunch of grapes’ (Dias et al., 2014; Veenvliet et al., 2020). The accessibility and scalability of stembryos makes them excellently suited to such chemical (but also genetic and mechanical) modulations in high-throughput, followed by multivariate feature analysis to obtain phenotypic fingerprints and infer the regulatory genetic interactions, as recently achieved in intestinal organoids (Lukonin et al., 2020). Adding a temporal component to the multivariate feature profiling will be important, especially in light of the finding that EBs do not develop synchronously (Boxman et al., 2016). In fact, the developmental time of stembryos (as opposed to culture time) may be an important determinant of the mechanical and/or chemical competence of cellular ensembles to external manipulations (e.g. CHIR pulse, Matrigel addition). Moreover, adding the fourth dimension (time) is essential to move beyond sole inference of regulatory genetic interactions as drivers of phenotypic variation, and incorporate the role of differential tissue mechanics.

## Conclusions and final remarks

An important rationale of stembryogenesis is that the ‘bottom-up’ approach allows us to ‘understand the whole from its parts’ (Cornwall-Scoones and Zernicka-Goetz, 2021; Gritti et al., 2021; Shahbazi et al., 2019). In this regard, a unique feature of stembryos is that models with different degrees of morphogenetic complexity, ranging from an elongated shape with established body axes but compromised morphology to a TLS, can be generated from the same starting material (i.e. pluripotent stem cell aggregates). As such, stembryos represent deconstructed embryos, in which various levels of architecture can be added by changing cell-intrinsic and -extrinsic determinants, using the original gastruloid protocol as a blank slate (Fig. 1). In addition, stembryos will be useful for improving our understanding of the design principles thought to be active in embryos (Fig. 3). As we have discussed, a direct comparative analysis of stembryos with different levels of morphological complexity may provide insights into the molecular, cellular and morphogenetic processes that shape the stembryo and, by proxy, the embryo. In addition, valuable insights may come from studying the developmental dynamics driving phenotypic variability; careful mapping of these routes not taken during normal development will teach us the constraints that act *in vivo* to ensure reproducible morphological outcomes and may help us to devise methods to make spontaneous variation controllable (Fig. 4).

To understand how (st)embryos take shape, we should move away from the idea that the decoding of gene regulatory programmes is sufficient to explain how cells form tissues. In multicellular organisms, cells do not act as isolated units, but are non-autonomous entities that integrate mechanical, biochemical and geometrical inputs in a bi-directional communication with their environment (Gorfinkiel and Martinez-Arias, 2021). Although many aspects of embryo morphogenesis may be triggered by genetic networks (programmed induction), most complex shapes are subsequently achieved through self-organised propagation (see discussion in Collinet and Lecuit, 2021). As we have discussed, global inputs can be reinforced at the cellular level to self-propagate organised tissue architecture, resulting in the robust sculpting of the mammalian embryo. Hence, to understand the molecular, cellular and morphogenetic principles that govern mammalian embryogenesis, studying the embryo *in toto* across spatial and temporal scales is pivotal. Indeed, such analysis has resulted in important insights from tractable and optically accessible species, such as *Xenopus* and zebrafish. However, the regulatory programmes and repertoire of cellular behaviours driving morphogenesis differ in mammalian embryos. For example, axial elongation dynamics differ between mouse and zebrafish, possibly related to differences in, for example, the landscape of mechanical forces and the more extensive coupling of growth and morphogenesis in mammalian embryos (Sambasivan and Steventon, 2020; Steventon et al., 2016; Sutherland, 2016). Thus, a detailed integrated analysis of mammalian post-implantation embryogenesis is needed. Finally, to move from correlation to causation, manipulating the feedback loop at the heart of embryo morphogenesis at all levels is crucial, for which stembryos provide a unique experimental platform. Importantly, a major recent technological advancement achieving *ex utero* culture of mouse embryos from pre-gastrulation to organogenesis stages could allow for testing concepts emerging from stembryos *in vivo*, even though throughput and accessibility is still limited compared with stembryos (Aguilera-Castrejon et al., 2021). Altogether, the recent advances in developmental engineering make the toolbox previously reserved for organoids and non-mammalian species applicable to the study of mammalian embryogenesis. Combining this toolbox with recent advances in biomedical engineering, imaging, image analysis, genomics and physical modelling will provide an unprecedented understanding of the developmental processes that sculpt the mammalian embryo in space and time.

## References

[DEV192914C1] Aguilera-Castrejon, A., Oldak, B., Shani, T., Ghanem, N., Itzkovich, C., Slomovich, S., Tarazi, S., Bayerl, J., Chugaeva, V., Ayyash, M. et al. (2021). Ex utero mouse embryogenesis from pre-gastrulation to late organogenesis. *Nature* 593, 119-124. 10.1038/s41586-021-03416-3 33731940

[DEV192914C2] Aisenbrey, E. A. and Murphy, W. L. (2020). Synthetic alternatives to Matrigel. *Nat Rev Mater.* 5, 539-551. 10.1038/s41578-020-0199-8 32953138 PMC7500703

[DEV192914C3] Alt, S., Ganguly, P. and Salbreux, G. (2017). Vertex models: from cell mechanics to tissue morphogenesis. *Philos. Trans. R. Soc. Lond. B, Biol. Sci.* 372, 20150520. 10.1098/rstb.2015.0520 28348254 PMC5379026

[DEV192914C4] Amadei, G., Lau, K. Y. C., De Jonghe, J., Gantner, C. W., Sozen, B., Chan, C., Zhu, M., Kyprianou, C., Hollfelder, F. and Zernicka-Goetz, M. (2021). Inducible stem-cell-derived embryos capture mouse morphogenetic events In Vitro. *Dev. Cell* 56, 366-382.e9. 10.1016/j.devcel.2020.12.004 33378662 PMC7883308

[DEV192914C5] Anlas, K. and Trivedi, V. (2021). Studying evolution of the primary body axis in vivo and in vitro. *Elife* 10, e69066. 10.7554/eLife.69066 34463611 PMC8456739

[DEV192914C6] Anlas, K., Gritti, N., Oriola, D., Arató, K., Nakaki, F., Lim, J. L., Sharpe, J. and Trivedi, V. (2021). Dynamics of anteroposterior axis establishment in a mammalian embryo-like system. *bioRxiv*. 10.1101/2021.02.24.432766

[DEV192914C8] Ashworth, J. C., Thompson, J. L., James, J. R., Slater, C. E., Pijuan-Galitó, S., Lis-Slimak, K., Holley, R. J., Meade, K. A., Thompson, A., Arkill, K. P. et al. (2020). Peptide gels of fully-defined composition and mechanics for probing cell-cell and cell-matrix interactions in vitro. *Matrix Biol.* 85-86, 15-33. 10.1016/j.matbio.2019.06.009 31295578 PMC7610915

[DEV192914C9] Baillie-Benson, P., Moris, N. and Martinez Arias, A. (2020). Pluripotent stem cell models of early mammalian development. *Curr. Opin. Cell Biol.* 66, 89-96. 10.1016/j.ceb.2020.05.010 32645551

[DEV192914C10] Bambardekar, K., Clément, R., Blanc, O., Chardès, C. and Lenne, P. F. (2015). Direct laser manipulation reveals the mechanics of cell contacts in vivo. *Proc. Natl. Acad. Sci. USA* 112, 1416-1421. 10.1073/pnas.1418732112 25605934 PMC4321260

[DEV192914C11] Barone, V., Lang, M., Krens, S. F. G., Pradhan, S. J., Shamipour, S., Sako, K., Sikora, M., Guet, C. C. and Heisenberg, C.-P. (2017). An effective feedback loop between cell-cell contact duration and morphogen signaling determines cell fate. *Dev. Cell* 43, 198-211.e12. 10.1016/j.devcel.2017.09.014 29033362

[DEV192914C12] Beccari, L., Moris, N., Girgin, M., Turner, D. A., Baillie-Johnson, P., Cossy, A.-C., Lutolf, M. P., Duboule, D. and Arias, A. M. (2018). Multi-axial self-organization properties of mouse embryonic stem cells into gastruloids. *Nature* 562, 272-276. 10.1038/s41586-018-0578-0 30283134

[DEV192914C13] Behrndt, M., Salbreux, G., Campinho, P., Hauschild, R., Oswald, F., Roensch, J., Grill, S. W. and Heisenberg, C.-P. (2012). Forces driving epithelial spreading in zebrafish gastrulation. *Science* 338, 257-260. 10.1126/science.1224143 23066079

[DEV192914C14] Belmonte, J. M., Leptin, M. and Nédélec, F. (2017). A theory that predicts behaviors of disordered cytoskeletal networks. *Mol. Syst. Biol.* 13, 941. 10.15252/msb.20177796 28954810 PMC5615920

[DEV192914C15] Bénazéraf, B., Francois, P., Baker, R. E., Denans, N., Little, C. D. and Pourquié, O. (2010). A random cell motility gradient downstream of FGF controls elongation of an amniote embryo. *Nature* 466, 248-252. 10.1038/nature09151 20613841 PMC3118990

[DEV192914C16] Bérenger-Currias, N. M. L. P., Mircea, M., Adegeest, E., van den Berg, P. R., Feliksik, M., Hochane, M., Idema, T., Tans, S. and Semrau, S. (2020). Early neurulation recapitulated in assemblies of embryonic and extraembryonic cells. *bioRxiv*. 10.1101/2020.02.13.947655

[DEV192914C17] Bi, D., Lopez, J. H., Schwarz, J. M. and Manning, M. L. (2015). A density-independent rigidity transition in biological tissues. *Nat. Phys.* 11, 1074-1079. 10.1038/nphys3471

[DEV192914C18] Blin, G., Wisniewski, D., Picart, C., Thery, M., Puceat, M. and Lowell, S. (2018). Geometrical confinement controls the asymmetric patterning of brachyury in cultures of pluripotent cells. *Development* 145, dev166025. 30115626 10.1242/dev.166025 PMC6176930

[DEV192914C19] Boxman, J., Sagy, N., Achanta, S., Vadigepalli, R. and Nachman, I. (2016). Integrated live imaging and molecular profiling of embryoid bodies reveals a synchronized progression of early differentiation. *Sci. Rep.* 6, 31623. 10.1038/srep31623 27530599 PMC4987683

[DEV192914C20] Brassard, J. A. and Lutolf, M. P. (2019). Engineering stem cell self-organization to build better organoids. *Cell Stem Cell* 24, 860-876. 10.1016/j.stem.2019.05.005 31173716

[DEV192914C21] Brizzi, M. F., Tarone, G. and Defilippi, P. (2012). Extracellular matrix, integrins, and growth factors as tailors of the stem cell niche. *Curr. Opin. Cell Biol.* 24, 645-651. 10.1016/j.ceb.2012.07.001 22898530

[DEV192914C22] Busby, L. and Steventon, B. (2021). Tissue tectonics and the multi-scale regulation of developmental timing. *Interface Focus* 11, 20200057. 10.1098/rsfs.2020.0057 34055304 PMC8086930

[DEV192914C23] Buske, P., Przybilla, J., Loeffler, M., Sachs, N., Sato, T., Clevers, H. and Galle, J. (2012). On the biomechanics of stem cell niche formation in the gut--modelling growing organoids. *FEBS J.* 279, 3475-3487. 10.1111/j.1742-4658.2012.08646.x 22632461

[DEV192914C24] Cachat, E., Liu, W., Martin, K. C., Yuan, X., Yin, H., Hohenstein, P. and Davies, J. A. (2016). 2- and 3-dimensional synthetic large-scale de novo patterning by mammalian cells through phase separation. *Sci. Rep.* 6, 20664. 10.1038/srep20664 26857385 PMC4746622

[DEV192914C25] Campàs, O. (2016). A toolbox to explore the mechanics of living embryonic tissues. *Semin. Cell Dev. Biol.* 55, 119-130. 10.1016/j.semcdb.2016.03.011 27061360 PMC4903887

[DEV192914C26] Campàs, O., Mammoto, T., Hasso, S., Sperling, R. A., O'Connell, D., Bischof, A. G., Maas, R., Weitz, D. A., Mahadevan, L. and Ingber, D. E. (2014). Quantifying cell-generated mechanical forces within living embryonic tissues. *Nat. Methods* 11, 183-189. 10.1038/nmeth.2761 24317254 PMC3939080

[DEV192914C27] Chan, C. E. and Odde, D. J. (2008). Traction dynamics of filopodia on compliant substrates. *Science* 322, 1687-1691. 10.1126/science.1163595 19074349

[DEV192914C28] Chan, C. J., Heisenberg, C.-P. and Hiiragi, T. (2017). Coordination of morphogenesis and cell-fate specification in development. *Curr. Biol.* 27, R1024-R1035. 10.1016/j.cub.2017.07.010 28950087

[DEV192914C29] Chiou, K. K., Hufnagel, L. and Shraiman, B. I. (2012). Mechanical stress inference for two dimensional cell arrays. *PLoS Comput. Biol.* 8, e1002512. 10.1371/journal.pcbi.1002512 22615550 PMC3355066

[DEV192914C30] Clément, R., Dehapiot, B., Collinet, C., Lecuit, T. and Lenne, P.-F. (2017). Viscoelastic dissipation stabilizes cell shape changes during tissue morphogenesis. *Curr. Biol.* 27, 3132-3142.e4. 10.1016/j.cub.2017.09.005 28988857

[DEV192914C31] Collinet, C. and Lecuit, T. (2021). Programmed and self-organized flow of information during morphogenesis. *Nat. Rev. Mol. Cell Biol.* 22, 245-265. 10.1038/s41580-020-00318-6 33483696

[DEV192914C32] Cornwall-Scoones, J. and Zernicka-Goetz, M. (2021). Unifying synthetic embryology. *Dev. Biol.* 474, 1-4. 10.1016/j.ydbio.2021.03.007 33753081 PMC8257322

[DEV192914C33] Dahl-Jensen, S. and Grapin-Botton, A. (2017). The physics of organoids: a biophysical approach to understanding organogenesis. *Development* 144, 946-951. 10.1242/dev.143693 28292839

[DEV192914C34] David, R., Ninomiya, H., Winklbauer, R. and Neumann, A. W. (2009). Tissue surface tension measurement by rigorous axisymmetric drop shape analysis. *Colloids Surf. B, Biointerfaces* 72, 236-240. 10.1016/j.colsurfb.2009.04.009 19442498

[DEV192914C35] Davidson, K. C., Mason, E. A. and Pera, M. F. (2015). The pluripotent state in mouse and human. *Development* 142, 3090-3099. 10.1242/dev.116061 26395138

[DEV192914C36] Davies, J. (2017). Using synthetic biology to explore principles of development. *Development* 144, 1146-1158. 10.1242/dev.144196 28351865

[DEV192914C37] de Medeiros, G., Ortiz, R., Strnad, P., Boni, A., Maurer, F. and Liberali, P. (2021). Multiscale light-sheet organoid imaging framework. *bioRxiv*. 10.1101/2021.05.12.443427 PMC9388485 35982061

[DEV192914C38] Dias, A. S., de Almeida, I., Belmonte, J. M., Glazier, J. A. and Stern, C. D. (2014). Somites without a clock. *Science* 343, 791-795. 10.1126/science.1247575 24407478 PMC3992919

[DEV192914C39] Doetschman, T. C., Eistetter, H., Katz, M., Schmidt, W. and Kemler, R. (1985). The in vitro development of blastocyst-derived embryonic stem cell lines: formation of visceral yolk sac, blood islands and myocardium. *J Embryol Exp Morphol* 87, 27-45. 3897439

[DEV192914C40] Durdu, S., Iskar, M., Revenu, C., Schieber, N., Kunze, A., Bork, P., Schwab, Y. and Gilmour, D. (2014). Luminal signalling links cell communication to tissue architecture during organogenesis. *Nature* 515, 120-124. 10.1038/nature13852 25337877

[DEV192914C41] Eiraku, M., Takata, N., Ishibashi, H., Kawada, M., Sakakura, E., Okuda, S., Sekiguchi, K., Adachi, T. and Sasai, Y. (2011). Self-organizing optic-cup morphogenesis in three-dimensional culture. *Nature* 472, 51-56. 10.1038/nature09941 21475194

[DEV192914C42] Erdemci-Tandogan, G. and Manning, M. L. (2021). Effect of cellular rearrangement time delays on the rheology of vertex models for confluent tissues. *PLoS Comput. Biol.* 17, e1009049. 10.1371/journal.pcbi.1009049 34097706 PMC8211246

[DEV192914C43] Etoc, F., Metzger, J., Ruzo, A., Kirst, C., Yoney, A., Ozair, M. Z., Brivanlou, A. H. and Siggia, E. D. (2016). A balance between secreted inhibitors and edge sensing controls gastruloid self-organization. *Dev. Cell* 39, 302-315. 10.1016/j.devcel.2016.09.016 27746044 PMC5113147

[DEV192914C44] Etournay, R., Merkel, M., Popović, M., Brandl, H., Dye, N. A., Aigouy, B., Salbreux, G., Eaton, S. and Jülicher, F. (2016). TissueMiner: A multiscale analysis toolkit to quantify how cellular processes create tissue dynamics. *Elife* 5, e14334. 10.7554/eLife.14334 27228153 PMC4946903

[DEV192914C45] Farhadifar, R., Röper, J.-C., Aigouy, B., Eaton, S. and Jülicher, F. (2007). The influence of cell mechanics, cell-cell interactions, and proliferation on epithelial packing. *Curr. Biol.* 17, 2095-2104. 10.1016/j.cub.2007.11.049 18082406

[DEV192914C46] Fletcher, A. G., Osterfield, M., Baker, R. E. and Shvartsman, S. Y. (2014). Vertex models of epithelial morphogenesis. *Biophys. J.* 106, 2291-2304. 10.1016/j.bpj.2013.11.4498 24896108 PMC4052277

[DEV192914C47] Forgacs, G., Foty, R. A., Shafrir, Y. and Steinberg, M. S. (1998). Viscoelastic properties of living embryonic tissues: a quantitative study. *Biophys. J.* 74, 2227-2234. 10.1016/S0006-3495(98)77932-9 9591650 PMC1299566

[DEV192914C48] Foty, R. A. and Steinberg, M. S. (2005). The differential adhesion hypothesis: a direct evaluation. *Dev. Biol.* 278, 255-263. 10.1016/j.ydbio.2004.11.012 15649477

[DEV192914C49] Fulton, T., Hwang, S., Wang, Y., Thomson, L., Clark, B., Verd, B. and Steventon, B. (2021). Morphogenetic coupling leads to pattern emergence in the pre somitic mesoderm. *bioRxiv*. 10.1101/2021.02.05.429898

[DEV192914C50] Gardel, M. L., Shin, J. H., MacKintosh, F. C., Mahadevan, L., Matsudaira, P. and Weitz, D. A. (2004). Elastic behavior of cross-linked and bundled actin networks. *Science* 304, 1301-1305. 10.1126/science.1095087 15166374

[DEV192914C51] Gharibi, B., Gonçalves, E., Nashun, B., Montoya, A., Mankalow, K., Strohbuecker, S., Sheriff, R. S. M., Cicarrelli, A., Carvalho, J., Nye, E. et al. (2020). A FGF2-mediated incoherent feedforward loop nduces Erk inhibition and promotes naïve pluripotency. *bioRxiv*. 10.1101/2020.11.11.378869

[DEV192914C52] Ghimire, S., Mantziou, V., Moris, N. and Martinez Arias, A. (2021). Human gastrulation: The embryo and its models. *Dev. Biol.* 474, 100-108. 10.1016/j.ydbio.2021.01.006 33484705

[DEV192914C53] Gierer, A. and Meinhardt, H. (1972). A theory of biological pattern formation. *Kybernetik* 12, 30-39. 10.1007/BF00289234 4663624

[DEV192914C54] Gillespie, D. T. (1977). Exact stochastic simulation of coupled chemical reactions. *J. Phys. Chem.* 81, 2340-2361. 10.1021/j100540a008

[DEV192914C55] Gilmour, D., Rembold, M. and Leptin, M. (2017). From morphogen to morphogenesis and back. *Nature* 541, 311-320. 10.1038/nature21348 28102269

[DEV192914C56] Girgin, M. U., Broguiere, N., Mattolini, L. and Lutolf, M. P. (2021a). Gastruloids generated without exogenous Wnt activation develop anterior neural tissues. *Stem Cell Rep* 16, 1143-1155. 10.1016/j.stemcr.2021.03.017 PMC8185432 33891872

[DEV192914C57] Girgin, M. U., Broguiere, N., Hoehnel, S., Brandenberg, N., Mercier, B., Arias, A. M. and Lutolf, M. P. (2021b). Bioengineered embryoids mimic post-implantation development in vitro. *Nat. Commun.* 12, 5140. 10.1038/s41467-021-25237-8 34446708 PMC8390504

[DEV192914C58] Girós, A., Grgur, K., Gossler, A. and Costell, M. (2011). α5β1 integrin-mediated adhesion to fibronectin is required for axis elongation and somitogenesis in mice. *PLoS One* 6, e22002. 10.1371/journal.pone.0022002 21799763 PMC3142108

[DEV192914C59] Gjorevski, N., Sachs, N., Manfrin, A., Giger, S., Bragina, M. E., Ordóñez-Morán, P., Clevers, H. and Lutolf, M. P. (2016). Designer matrices for intestinal stem cell and organoid culture. *Nature* 539, 560-564. 10.1038/nature20168 27851739

[DEV192914C60] Gómez-González, M., Latorre, E., Arroyo, M. and Trepat, X. (2020). Measuring mechanical stress in living tissues. *Nat. Rev. Phys.* 2, 300-317. 10.1038/s42254-020-0184-6 PMC7617344 39867749

[DEV192914C61] Gorfinkiel, N. and Martinez-Arias, A. (2021). The cell in the age of the genomic revolution: cell regulatory networks. *Cells Dev.* 203720. 10.1016/j.cdev.2021.203720 34252599

[DEV192914C62] Grill, S. W., Gönczy, P., Stelzer, E. H. and Hyman, A. A. (2001). Polarity controls forces governing asymmetric spindle positioning in the Caenorhabditis elegans embryo. *Nature* 409, 630-633. 10.1038/35054572 11214323

[DEV192914C63] Gritti, N., Oriola, D. and Trivedi, V. (2021). Rethinking embryology in vitro: A synergy between engineering, data science and theory. *Dev. Biol.* 474, 48-61. 10.1016/j.ydbio.2020.10.013 33152275

[DEV192914C64] Guevorkian, K., Colbert, M.-J., Durth, M., Dufour, S. and Brochard-Wyart, F. (2010). Aspiration of biological viscoelastic drops. *Phys. Rev. Lett.* 104, 218101. 10.1103/PhysRevLett.104.218101 20867138

[DEV192914C65] Gupta, A., Lutolf, M. P., Hughes, A. J. and Sonnen, K. F. (2021). Bioengineering in vitro models of embryonic development. *Stem Cell Rep.* 16, 1104-1116. 10.1016/j.stemcr.2021.04.005 PMC8185467 33979597

[DEV192914C66] Hammerschmidt, M. and Wedlich, D. (2008). Regulated adhesion as a driving force of gastrulation movements. *Development* 135, 3625-3641. 10.1242/dev.015701 18952908

[DEV192914C67] Hannezo, E. and Heisenberg, C.-P. (2019). Mechanochemical feedback loops in development and disease. *Cell* 178, 12-25. 10.1016/j.cell.2019.05.052 31251912

[DEV192914C68] Hannezo, E., Prost, J. and Joanny, J.-F. (2014). Theory of epithelial sheet morphology in three dimensions. *Proc. Natl. Acad. Sci. USA* 111, 27-32. 10.1073/pnas.1312076111 24367079 PMC3890844

[DEV192914C69] Harrison, S. E., Sozen, B., Christodoulou, N., Kyprianou, C. and Zernicka-Goetz, M. (2017). Assembly of embryonic and extraembryonic stem cells to mimic embryogenesis in vitro. *Science* 356, eaal1810. 10.1126/science.aal1810 28254784

[DEV192914C70] Hartmann, J., Krueger, D. and De Renzis, S. (2020). Using optogenetics to tackle systems-level questions of multicellular morphogenesis. *Curr. Opin. Cell Biol.* 66, 19-27. 10.1016/j.ceb.2020.04.004 32408249

[DEV192914C71] Hashmi, A., Tlili, S., Perrin, P., Martinez Arias, A. and Lenne, P.-F. (2020). Cell state transitions and collective cell movement generate an endoderm-like region in gastruloids. *bioRxiv*. 10.1101/2020.05.21.105551 PMC9033300 35404233

[DEV192914C72] He, Z., Gerber, T., Maynard, A., Jain, A., Petri, R., Santel, M., Ly, K., Sidow, L., Sanchis Calleja, F., Riesenberg, S. et al. (2020). Lineage recording reveals dynamics of cerebral organoid regionalization. *bioRxiv*. 10.1101/2020.06.19.162032

[DEV192914C73] Hiramatsu, R., Matsuoka, T., Kimura-Yoshida, C., Han, S.-W., Mochida, K., Adachi, T., Takayama, S. and Matsuo, I. (2013). External mechanical cues trigger the establishment of the anterior-posterior axis in early mouse embryos. *Dev. Cell* 27, 131-144. 10.1016/j.devcel.2013.09.026 24176640

[DEV192914C74] Hughes, C. S., Postovit, L. M. and Lajoie, G. A. (2010). Matrigel: a complex protein mixture required for optimal growth of cell culture. *Proteomics* 10, 1886-1890. 10.1002/pmic.200900758 20162561

[DEV192914C75] Hwang, Y.-S., Chung, B. G., Ortmann, D., Hattori, N., Moeller, H.-C. and Khademhosseini, A. (2009). Microwell-mediated control of embryoid body size regulates embryonic stem cell fate via differential expression of WNT5a and WNT11. *Proc. Natl. Acad. Sci. USA* 106, 16978-16983. 10.1073/pnas.0905550106 19805103 PMC2761314

[DEV192914C76] Ishihara, K. and Tanaka, E. M. (2018). Spontaneous symmetry breaking and pattern formation of organoids. *Current Opinion in Systems Biology* 11, 123-128. 10.1016/j.coisb.2018.06.002

[DEV192914C187] Itskovitz-Eldor, J., Schuldiner, M., Karsenti, D., Eden, A., Yanuka, O., Amit, M., Soreq, H. and Benvenisty, N. (2000). Differentiation of human embryonic stem cells into embryoid bodies compromising the three embryonic germ layers. *Mol. Med.* 6, 88-95. 10859025 PMC1949933

[DEV192914C77] Jabaudon, D. and Lancaster, M. (2018). Exploring landscapes of brain morphogenesis with organoids. *Development* 145, dev172049. 10.1242/dev.172049 30455367

[DEV192914C78] Jakab, K., Damon, B., Marga, F., Doaga, O., Mironov, V., Kosztin, I., Markwald, R. and Forgacs, G. (2008). Relating cell and tissue mechanics: implications and applications. *Dev. Dyn.* 237, 2438-2449. 10.1002/dvdy.21684 18729216

[DEV192914C79] Jilkine, A., Marée, A. F. M. and Edelstein-Keshet, L. (2007). Mathematical model for spatial segregation of the Rho-family GTPases based on inhibitory crosstalk. *Bull. Math. Biol.* 69, 1943-1978. 10.1007/s11538-007-9200-6 17457653

[DEV192914C80] Kim, S., Pochitaloff, M., Stooke-Vaughan, G. A. and Campàs, O. (2021). Embryonic tissues as active foams. *Nat. Phys* 17, 859-866. 10.1038/s41567-021-01215-1 34367313 PMC8336761

[DEV192914C81] Kirillova, A., Genikhovich, G., Pukhlyakova, E., Demilly, A., Kraus, Y. and Technau, U. (2018). Germ-layer commitment and axis formation in sea anemone embryonic cell aggregates. *Proc. Natl. Acad. Sci. USA* 115, 1813-1818. 10.1073/pnas.1711516115 29440382 PMC5828576

[DEV192914C82] Kleinman, H. K. and Martin, G. R. (2005). Matrigel: basement membrane matrix with biological activity. *Semin. Cancer Biol.* 15, 378-386. 10.1016/j.semcancer.2005.05.004 15975825

[DEV192914C83] Klopper, A. V., Krens, G., Grill, S. W. and Heisenberg, C. P. (2010). Finite-size corrections to scaling behavior in sorted cell aggregates. *Eur. Phys. J. E Soft Matter* 33, 99-103. 10.1140/epje/i2010-10642-y 20852912

[DEV192914C84] Krieg, M., Arboleda-Estudillo, Y., Puech, P. H., Käfer, J., Graner, F., Müller, D. J. and Heisenberg, C. P. (2008). Tensile forces govern germ-layer organization in zebrafish. *Nat. Cell Biol.* 10, 429-436. 10.1038/ncb1705 18364700

[DEV192914C85] Kumar, A., Placone, J. K. and Engler, A. J. (2017). Understanding the extracellular forces that determine cell fate and maintenance. *Development* 144, 4261-4270. 10.1242/dev.158469 29183939 PMC5769638

[DEV192914C86] Kunz, D., Wang, A., Chan, C. U., Pritchard, R. H., Wang, W., Gallo, F., Bradshaw, C. R., Terenzani, E., Müller, K. H., Huang, Y. Y. S. et al. (2021). Downregulation of extraembryonic tension controls body axis formation in avian embryos. *bioRxiv*. 10.1101/2021.02.24.432525 PMC10241863 37277340

[DEV192914C87] Kyprianou, C., Christodoulou, N., Hamilton, R. S., Nahaboo, W., Boomgaard, D. S., Amadei, G., Migeotte, I. and Zernicka-Goetz, M. (2020). Basement membrane remodelling regulates mouse embryogenesis. *Nature* 582, 253-258. 10.1038/s41586-020-2264-2 32523119 PMC7308173

[DEV192914C88] Leahy, A., Xiong, J. W., Kuhnert, F. and Stuhlmann, H. (1999). Use of developmental marker genes to define temporal and spatial patterns of differentiation during embryoid body formation. *J. Exp. Zool.* 284, 67-81. 10.1002/(SICI)1097-010X(19990615)284:1<67::AID-JEZ10>3.0.CO;2-O 10368935

[DEV192914C89] Lebreton, G., Géminard, C., Lapraz, F., Pyrpassopoulos, S., Cerezo, D., Spéder, P., Ostap, E. M. and Noselli, S. (2018). Molecular to organismal chirality is induced by the conserved myosin 1D. *Science* 362, 949-952. 10.1126/science.aat8642 30467170 PMC6698710

[DEV192914C90] Lenne, P.-F. and Trivedi, V. (2018). Tissue “melting” sculpts embryo. *Nature* 561, 315-316. 10.1038/d41586-018-06108-7 30224727

[DEV192914C91] Lenne, P.-F. and Trivedi, V. (2021). Sculpting tissues by phase transitions. *Nat Commun.* (in press). 10.1038/s41467-022-28151-9 PMC8814027 35115507

[DEV192914C92] Lenne, P.-F., Munro, E., Heemskerk, I., Warmflash, A., Bocanegra-Moreno, L., Kishi, K., Kicheva, A., Long, Y., Fruleux, A., Boudaoud, A. et al. (2021). Roadmap for the multiscale coupling of biochemical and mechanical signals during development. *Phys. Biol.* 18, 041501. 10.1088/1478-3975/abd0db PMC8380410 33276350

[DEV192914C93] Libby, A. R. G., Joy, D. A., Elder, N. H., Bulger, E. A., Krakora, M. Z., Gaylord, E. A., Mendoza-Camacho, F., Butts, J. C. and McDevitt, T. C. (2021). Axial elongation of caudalized human organoids mimics aspects of neural tube development. *Development* 148, dev198275. 10.1242/dev.198275 34142711 PMC8254868

[DEV192914C94] Liu, L. and Warmflash, A. (2021). Self-organized signaling in stem cell models of embryos. *Stem Cell Reports* 16, 1065-1077. 10.1016/j.stemcr.2021.03.020 33979594 PMC8185436

[DEV192914C95] López-Anguita, N., Gassaloglu, S. A., Stötzel, M., Typou, M., Virta, I., Hetzel, S., Buschow, R., Koksal, B., Atilla, D., Maitschke-Rajasekharan, R. et al. (2021). Hypoxia induces a transcriptional early primitive streak signature in pluripotent cells enhancing spontaneous elongation and lineage segregation in gastruloids. *bioRxiv*. 10.1101/2021.07.21.452906

[DEV192914C96] Lukonin, I., Serra, D., Challet Meylan, L., Volkmann, K., Baaten, J., Zhao, R., Meeusen, S., Colman, K., Maurer, F., Stadler, M. B. et al. (2020). Phenotypic landscape of intestinal organoid regeneration. *Nature* 586, 275-280. 10.1038/s41586-020-2776-9 33029001 PMC7116869

[DEV192914C97] Manfrin, A., Tabata, Y., Paquet, E. R., Vuaridel, A. R., Rivest, F. R., Naef, F. and Lutolf, M. P. (2019). Engineered signaling centers for the spatially controlled patterning of human pluripotent stem cells. *Nat. Methods* 16, 640-648. 10.1038/s41592-019-0455-2 31249412

[DEV192914C98] Marikawa, Y., Tamashiro, D. A. A., Fujita, T. C. and Alarcón, V. B. (2009). Aggregated P19 mouse embryonal carcinoma cells as a simple in vitro model to study the molecular regulations of mesoderm formation and axial elongation morphogenesis. *Genesis* 47, 93-106. 10.1002/dvg.20473 19115346 PMC3419260

[DEV192914C188] Marikawa, Y., Chen, H. R., Menor, M., Deng, Y. and Alarcon, V. B. (2020). Exposure-based assessment of chemical teratogenicity using morphogenetic aggregates of human embryonic stem cells. *Reprod Toxicol.* 91, 74-91. 10.1016/j.reprotox.2019.10.004 31711903 PMC6980740

[DEV192914C99] Martinez-Ara, G., Taberner, N., Takayama, M., Sandaltzopoulou, E., Villava, C. E., Takata, N., Eiraku, M. and Ebisuya, M. (2021). Optogenetic control of apical constriction induces synthetic morphogenesis in mammalian tissues. *bioRxiv*. 10.1101/2021.04.20.440475 PMC9474505 36104355

[DEV192914C100] Matsuo, I. and Hiramatsu, R. (2017). Mechanical perspectives on the anterior-posterior axis polarization of mouse implanted embryos. *Mech. Dev.* 144, 62-70. 10.1016/j.mod.2016.09.002 27697519

[DEV192914C101] Matthews, K. R. W., Wagner, D. S. and Warmflash, A. (2021). Stem cell-based models of embryos: the need for improved naming conventions. *Stem Cell Reports* 16, 1014-1020. 10.1016/j.stemcr.2021.02.018 33770498 PMC8185370

[DEV192914C102] McDole, K., Guignard, L., Amat, F., Berger, A., Malandain, G., Royer, L. A., Turaga, S. C., Branson, K. and Keller, P. J. (2018). In toto imaging and reconstruction of post-implantation mouse development at the single-cell level. *Cell* 175, 859-876.e33. 10.1016/j.cell.2018.09.031 30318151

[DEV192914C103] Meinhardt, H. (2008). Models of biological pattern formation: from elementary steps to the organization of embryonic axes. *Curr. Top. Dev. Biol.* 81, 1-63. 10.1016/S0070-2153(07)81001-5 18023723

[DEV192914C189] Meinhardt, A., Eberle, D., Tazaki, A., Ranga, A., Niesche, M., Wilsch-Bräuninger, M., Stec, A., Schackert, G., Lutolf, M. and Tanaka, E. M. (2014). 3D reconstitution of the patterned neural tube from embryonic stem cells. *Stem Cell Reports* 3, 987-999. 10.1016/j.stemcr.2014.09.020 25454634 PMC4264068

[DEV192914C104] Merkel, M., Etournay, R., Popović, M., Salbreux, G., Eaton, S. and Jülicher, F. (2017). Triangles bridge the scales: Quantifying cellular contributions to tissue deformation. *Phys. Rev. E* 95, 032401. 10.1103/PhysRevE.95.032401 28415200

[DEV192914C105] Minn, K. T., Fu, Y. C., He, S., Dietmann, S., George, S. C., Anastasio, M. A., Morris, S. A. and Solnica-Krezel, L. (2020). High-resolution transcriptional and morphogenetic profiling of cells from micropatterned human ESC gastruloid cultures. *Elife* 9, e59445. 10.7554/eLife.59445 33206048 PMC7728446

[DEV192914C106] Mohagheghian, E., Luo, J., Chen, J., Chaudhary, G., Chen, J., Sun, J., Ewoldt, R. H. and Wang, N. (2018). Quantifying compressive forces between living cell layers and within tissues using elastic round microgels. *Nat. Commun.* 9, 1878. 10.1038/s41467-018-04245-1 29760452 PMC5951850

[DEV192914C107] Molè, M. A., Galea, G. L., Rolo, A., Weberling, A., Nychyk, O., De Castro, S. C., Savery, D., Fässler, R., Ybot-González, P., Greene, N. D. E. et al. (2020). Integrin-mediated focal anchorage drives epithelial zippering during mouse neural tube closure. *Dev. Cell* 52, 321-334.e6. 10.1016/j.devcel.2020.01.012 32049039 PMC7008250

[DEV192914C108] Mongera, A., Rowghanian, P., Gustafson, H. J., Shelton, E., Kealhofer, D. A., Carn, E. K., Serwane, F., Lucio, A. A., Giammona, J. and Campàs, O. (2018). A fluid-to-solid jamming transition underlies vertebrate body axis elongation. *Nature* 561, 401-405. 10.1038/s41586-018-0479-2 30185907 PMC6148385

[DEV192914C109] Mori, Y., Jilkine, A. and Edelstein-Keshet, L. (2008). Wave-pinning and cell polarity from a bistable reaction-diffusion system. *Biophys. J.* 94, 3684-3697. 10.1529/biophysj.107.120824 18212014 PMC2292363

[DEV192914C110] Moris, N., Anlas, K., van den Brink, S. C., Alemany, A., Schröder, J., Ghimire, S., Balayo, T., van Oudenaarden, A. and Martinez Arias, A. (2020). An in vitro model of early anteroposterior organization during human development. *Nature* 582, 410-415. 10.1038/s41586-020-2383-9 32528178

[DEV192914C111] Moris, N., Alev, C., Pera, M. and Martinez Arias, A. (2021). Biomedical and societal impacts of in vitro embryo models of mammalian development. *Stem Cell Rep.* 16, 1021-1030. 10.1016/j.stemcr.2021.03.023 PMC8185435 33979591

[DEV192914C112] Mumford, T. R., Roth, L. and Bugaj, L. J. (2020). Reverse and forward engineering multicellular structures with optogenetics. *Current Opinion in Biomedical Engineering* 16, 61-71. 10.1016/j.cobme.2020.100250 33718689 PMC7945718

[DEV192914C113] Muncie, J. M., Ayad, N. M. E., Lakins, J. N., Xue, X., Fu, J. and Weaver, V. M. (2020). Mechanical tension promotes formation of gastrulation-like nodes and patterns mesoderm specification in human embryonic stem cells. *Dev. Cell* 55, 679-694.e11. 10.1016/j.devcel.2020.10.015 33207224 PMC7755684

[DEV192914C114] Muñoz-Descalzo, S., Hadjantonakis, A.-K. and Arias, A. M. (2015). Wnt/ß-catenin signalling and the dynamics of fate decisions in early mouse embryos and embryonic stem (ES) cells. *Semin. Cell Dev. Biol.* 47-48, 101-109. 10.1016/j.semcdb.2015.08.011 26321498 PMC4955857

[DEV192914C115] Münster, S., Jain, A., Mietke, A., Pavlopoulos, A., Grill, S. W. and Tomancak, P. (2019). Attachment of the blastoderm to the vitelline envelope affects gastrulation of insects. *Nature* 568, 395-399. 10.1038/s41586-019-1044-3 30918398

[DEV192914C116] Murisic, N., Hakim, V., Kevrekidis, I. G., Shvartsman, S. Y. and Audoly, B. (2015). From discrete to continuum models of three-dimensional deformations in epithelial sheets. *Biophys. J.* 109, 154-163. 10.1016/j.bpj.2015.05.019 26153712 PMC4571022

[DEV192914C117] Ninomiya, H., David, R., Damm, E. W., Fagotto, F., Niessen, C. M. and Winklbauer, R. (2012). Cadherin-dependent differential cell adhesion in Xenopus causes cell sorting in vitro but not in the embryo. *J. Cell Sci.* 125, 1877-1883. 22328523 10.1242/jcs.095315

[DEV192914C118] Okuda, S., Takata, N., Hasegawa, Y., Kawada, M., Inoue, Y., Adachi, T., Sasai, Y. and Eiraku, M. (2018). Strain-triggered mechanical feedback in self-organizing optic-cup morphogenesis. *Sci. Adv.* 4, eaau1354. 10.1126/sciadv.aau1354 30474058 PMC6248953

[DEV192914C119] Ollé-Vila, A., Duran-Nebreda, S., Conde-Pueyo, N., Montañez, R. and Solé, R. (2016). A morphospace for synthetic organs and organoids: the possible and the actual. *Integr Biol (Camb)* 8, 485-503. 10.1039/C5IB00324E 27032985

[DEV192914C120] Olmsted, Z. T. and Paluh, J. L. (2021). Co-development of central and peripheral neurons with trunk mesendoderm in human elongating multi-lineage organized gastruloids. *Nat. Commun.* 12, 3020. 10.1038/s41467-021-23294-7 34021144 PMC8140076

[DEV192914C121] Oriola, D., Marin-Riera, M., Aalderink, G., Anlas, K., Gritti, N., Sharpe, J. and Trivedi, V. (2020). Arrested coalescence of multicellular aggregates. *arXiv*. 2012.01455 [cond-mat.soft]. 10.1039/d2sm00063f 35511111

[DEV192914C122] Ortmann, D., Brown, S., Czechanski, A., Aydin, S., Muraro, D., Huang, Y., Tomaz, R. A., Osnato, A., Canu, G., Wesley, B. T. et al. (2020). Naive pluripotent stem cells exhibit phenotypic variability that is driven by genetic variation. *Cell Stem Cell* 27, 470-481.e6. 10.1016/j.stem.2020.07.019 32795399 PMC7487768

[DEV192914C123] Parker, A., Marchetti, M. C., Manning, M. L. and Schwarz, J. M. (2020). How does the extracellular matrix affect the rigidity of an embedded spheroid? *arXiv*. 2006.16203v1 [q-bio.CB] 10.1103/PhysRevE.111.044410 40411055

[DEV192914C124] Pera, M. F. (2017). Human embryo research and the 14-day rule. *Development* 144, 1923-1925. 10.1242/dev.151191 28559237

[DEV192914C125] Petridou, N. I. and Heisenberg, C.-P. (2019). Tissue rheology in embryonic organization. *EMBO J.* 38, e102497. 10.15252/embj.2019102497 31512749 PMC6792012

[DEV192914C126] Petridou, N. I., Grigolon, S., Salbreux, G., Hannezo, E. and Heisenberg, C.-P. (2019). Fluidization-mediated tissue spreading by mitotic cell rounding and non-canonical Wnt signalling. *Nat. Cell Biol.* 21, 169-178. 10.1038/s41556-018-0247-4 30559456

[DEV192914C127] Petridou, N. I., Corominas-Murtra, B., Heisenberg, C.-P. and Hannezo, E. (2021). Rigidity percolation uncovers a structural basis for embryonic tissue phase transitions. *Cell* 184, 1914-1928.e19. 10.1016/j.cell.2021.02.017 33730596 PMC8055543

[DEV192914C128] Pour, M., Kumar, A. S., Walther, M., Wittler, L., Meissner, A. and Nachman, I. (2019). Emergence and patterning dynamics of mouse definitive endoderm. *bioRxiv*. 10.1101/728642 PMC8693470 34988400

[DEV192914C129] Ranga, A., Girgin, M., Meinhardt, A., Eberle, D., Caiazzo, M., Tanaka, E. M. and Lutolf, M. P. (2016). Neural tube morphogenesis in synthetic 3D microenvironments. *Proc. Natl. Acad. Sci. USA* 113, E6831-E6839. 10.1073/pnas.1603529113 27742791 PMC5098636

[DEV192914C130] Repina, N. A., Bao, X., Zimmermann, J. A., Joy, D. A., Kane, R. S. and Schaffer, D. V. (2019). Optogenetic control of Wnt signaling for modeling early embryogenic patterning with human pluripotent stem cells. *bioRxiv*. 10.1101/665695 PMC10399980 37401411

[DEV192914C131] Rivron, N. C., Frias-Aldeguer, J., Vrij, E. J., Boisset, J. C., Korving, J., Vivié, J., Truckenmüller, R. K., van Oudenaarden, A., van Blitterswijk, C. A. and Geijsen, N. (2018). Blastocyst-like structures generated solely from stem cells. *Nature* 557, 106-111. 10.1038/s41586-018-0051-0 29720634

[DEV192914C132] Rossi, G., Broguiere, N., Miyamoto, M., Boni, A., Guiet, R., Girgin, M., Kelly, R. G., Kwon, C. and Lutolf, M. P. (2021). Capturing cardiogenesis in gastruloids. *Cell Stem Cell* 28, 230-240.e6. 10.1016/j.stem.2020.10.013 33176168 PMC7867643

[DEV192914C133] Royer, C., Leonavicius, K., Kip, A., Fortin, D., Nandi, K., Vincent, A., Jones, C., Child, T., Coward, K., Graham, C. et al. (2020). Establishment of a relationship between blastomere geometry and YAP localisation during compaction. *Development* 147, dev189449. 10.1242/dev.189449 32928909 PMC7561472

[DEV192914C134] Rozario, T. and DeSimone, D. W. (2010). The extracellular matrix in development and morphogenesis: a dynamic view. *Dev. Biol.* 341, 126-140. 10.1016/j.ydbio.2009.10.026 19854168 PMC2854274

[DEV192914C135] Sagy, N., Slovin, S., Allalouf, M., Pour, M., Savyon, G., Boxman, J. and Nachman, I. (2019). Prediction and control of symmetry breaking in embryoid bodies by environment and signal integration. *Development* 146, dev181917. 10.1242/dev.181917 31575644

[DEV192914C136] Samal, P., Maurer, P., van Blitterswijk, C., Truckenmüller, R. and Giselbrecht, S. (2020). A new microengineered platform for 4D tracking of single cells in a stem-cell-based In Vitro morphogenesis model. *Adv. Mater.* 32, e1907966. 10.1002/adma.201907966 32346909

[DEV192914C137] Sambasivan, R. and Steventon, B. (2020). Neuromesodermal progenitors: a basis for robust axial patterning in development and evolution. *Front. Cell Dev. Biol.* 8, 607516. 10.3389/fcell.2020.607516 33520989 PMC7843932

[DEV192914C138] Sánchez, A., Jones, W. K., Gulick, J., Doetschman, T. and Robbins, J. (1991). Myosin heavy chain gene expression in mouse embryoid bodies. An in vitro developmental study. *J. Biol. Chem.* 266, 22419-22426. 10.1016/S0021-9258(18)54589-0 1939265

[DEV192914C139] Sanematsu, P. C., Erdemci-Tandogan, G., Patel, H., Retzlaff, E. M., Amack, J. D. and Manning, M. L. (2021). 3D viscoelastic drag forces contribute to cell shape changes during organogenesis in the zebrafish embryo. *Cells Dev.* 203718. 10.1016/j.cdev.2021.203718 34273601 PMC8758797

[DEV192914C140] Schauer, A. and Heisenberg, C.-P. (2021). Reassembling gastrulation. *Dev. Biol.* 474, 71-81. 10.1016/j.ydbio.2020.12.014 33352181

[DEV192914C141] Serra, D., Mayr, U., Boni, A., Lukonin, I., Rempfler, M., Challet Meylan, L., Stadler, M. B., Strnad, P., Papasaikas, P., Vischi, D. et al. (2019). Self-organization and symmetry breaking in intestinal organoid development. *Nature* 569, 66-72. 10.1038/s41586-019-1146-y 31019299 PMC6544541

[DEV192914C142] Serwane, F., Mongera, A., Rowghanian, P., Kealhofer, D. A., Lucio, A. A., Hockenbery, Z. M. and Campàs, O. (2017). In vivo quantification of spatially varying mechanical properties in developing tissues. *Nat. Methods* 14, 181-186. 10.1038/nmeth.4101 27918540 PMC5524219

[DEV192914C143] Shah, G., Thierbach, K., Schmid, B., Waschke, J., Reade, A., Hlawitschka, M., Roeder, I., Scherf, N. and Huisken, J. (2019). Multi-scale imaging and analysis identify pan-embryo cell dynamics of germlayer formation in zebrafish. *Nat. Commun.* 10, 5753. 10.1038/s41467-019-13625-0 31848345 PMC6917746

[DEV192914C144] Shahbazi, M. N. and Zernicka-Goetz, M. (2018). Deconstructing and reconstructing the mouse and human early embryo. *Nat. Cell Biol.* 20, 878-887. 10.1038/s41556-018-0144-x 30038253

[DEV192914C145] Shahbazi, M. N., Siggia, E. D. and Zernicka-Goetz, M. (2019). Self-organization of stem cells into embryos: A window on early mammalian development. *Science* 364, 948-951. 10.1126/science.aax0164 31171690 PMC8300856

[DEV192914C146] Sharpe, J. (2017). Computer modeling in developmental biology: growing today, essential tomorrow. *Development* 144, 4214-4225. 10.1242/dev.151274 29183935

[DEV192914C147] Shelton, E. R., Kim, S., Gross, B. J., Wu, R., Pochitaloff, M., Lim, I., Sletten, E. M. and Campàs, O. (2021). Stress-driven tissue fluidization physically segments vertebrate somites. *bioRxiv*. 10.1101/2021.03.27.437325

[DEV192914C148] Shiri, Z., Simorgh, S., Naderi, S. and Baharvand, H. (2019). Optogenetics in the era of cerebral organoids. *Trends Biotechnol.* 37, 1282-1294. 10.1016/j.tibtech.2019.05.009 31227305

[DEV192914C149] Shyer, A. E., Huycke, T. R., Lee, C., Mahadevan, L. and Tabin, C. J. (2015). Bending gradients: how the intestinal stem cell gets its home. *Cell* 161, 569-580. 10.1016/j.cell.2015.03.041 25865482 PMC4409931

[DEV192914C192] Simunovic, M., Metzger, J. J., Etoc, F., Yoney, A., Ruzo, A., Martyn, I., Croft, G., You, D. S., Brivanlou, A. H. and Siggia, E. D. (2019). A 3D model of a human epiblast reveals BMP4-driven symmetry breaking. *Nat. Cell Biol.* 21, 900-910. 10.1038/s41556-019-0349-7 31263269

[DEV192914C150] Solnica-Krezel, L. and Sepich, D. S. (2012). Gastrulation: making and shaping germ layers. *Annu. Rev. Cell Dev. Biol.* 28, 687-717. 10.1146/annurev-cellbio-092910-154043 22804578

[DEV192914C151] Sonnen, K. F. and Aulehla, A. (2014). Dynamic signal encoding—from cells to organisms. *Semin. Cell Dev. Biol.* 34, 91-98. 10.1016/j.semcdb.2014.06.019 25008461

[DEV192914C152] Sozen, B., Amadei, G., Cox, A., Wang, R., Na, E., Czukiewska, S., Chappell, L., Voet, T., Michel, G., Jing, N. et al. (2018). Self-assembly of embryonic and two extra-embryonic stem cell types into gastrulating embryo-like structures. *Nat. Cell Biol.* 20, 979-989. 10.1038/s41556-018-0147-7 30038254

[DEV192914C153] Stapornwongkul, K. S. and Vincent, J.-P. (2021). Generation of extracellular morphogen gradients: the case for diffusion. *Nat. Rev. Genet* 22, 393-411. 10.1038/s41576-021-00342-y 33767424

[DEV192914C154] Stapornwongkul, K. S., de Gennes, M., Cocconi, L., Salbreux, G. and Vincent, J.-P. (2020). Patterning and growth control in vivo by an engineered GFP gradient. *Science* 370, 321-327. 10.1126/science.abb8205 33060356 PMC7611032

[DEV192914C155] Steinberg, M. S. (1970). Does differential adhesion govern self-assembly processes in histogenesis? Equilibrium configurations and the emergence of a hierarchy among populations of embryonic cells. *J. Exp. Zool.* 173, 395-433. 10.1002/jez.1401730406 5429514

[DEV192914C156] Steventon, B., Duarte, F., Lagadec, R., Mazan, S., Nicolas, J.-F. and Hirsinger, E. (2016). Species-specific contribution of volumetric growth and tissue convergence to posterior body elongation in vertebrates. *Development* 143, 1732-1741. 26989170 10.1242/dev.126375

[DEV192914C157] Streichan, S. J., Lefebvre, M. F., Noll, N., Wieschaus, E. F. and Shraiman, B. I. (2018). Global morphogenetic flow is accurately predicted by the spatial distribution of myosin motors. *Elife* 7, e27454. 10.7554/eLife.27454 29424685 PMC5843464

[DEV192914C158] Sugimura, K., Lenne, P.-F. and Graner, F. (2016). Measuring forces and stresses in situ in living tissues. *Development* 143, 186-196. 10.1242/dev.119776 26786209

[DEV192914C159] Sutherland, A. E. (2016). Tissue morphodynamics shaping the early mouse embryo. *Semin. Cell Dev. Biol.* 55, 89-98. 10.1016/j.semcdb.2016.01.033 26820524

[DEV192914C160] Ten Berge, D., Koole, W., Fuerer, C., Fish, M., Eroglu, E. and Nusse, R. (2008). Wnt signaling mediates self-organization and axis formation in embryoid bodies. *Cell Stem Cell* 3, 508-518. 10.1016/j.stem.2008.09.013 18983966 PMC2683270

[DEV192914C161] Thalheim, T., Quaas, M., Herberg, M., Braumann, U.-D., Kerner, C., Loeffler, M., Aust, G. and Galle, J. (2018). Linking stem cell function and growth pattern of intestinal organoids. *Dev. Biol.* 433, 254-261. 10.1016/j.ydbio.2017.10.013 29198564

[DEV192914C190] Thomson, L., Muresan, L. and Steventon, B. (2021). The zebrafish presomitic mesoderm elongates through compaction-extension. *Cells Dev.* 203748. 10.1016/j.cdev.2021.203748 PMC7612712 34597846

[DEV192914C163] Toda, S., McKeithan, W. L., Hakkinen, T. J., Lopez, P., Klein, O. D. and Lim, W. A. (2020). Engineering synthetic morphogen systems that can program multicellular patterning. *Science* 370, 327-331. 10.1126/science.abc0033 33060357 PMC7986291

[DEV192914C191] Torregrosa, G. and Garcia-Ojalvo, J. (2021). Mechanistic models of cell-fate transitions from single-cell data. *Curr. Opin. Syst. Biol.* 26, 79-86. 10.1016/j.coisb.2021.04.004

[DEV192914C165] Townes, P. L. and Holtfreter, J. (1955). Directed movements and selective adhesion of embryonic amphibian cells. *J. Exp. Zool.* 128, 53-120. 10.1002/jez.1401280105 15559931

[DEV192914C166] Trepat, X. and Sahai, E. (2018). Mesoscale physical principles of collective cell organization. *Nat. Phys.* 14, 671-682. 10.1038/s41567-018-0194-9

[DEV192914C167] Turing, A. M. (1952). The chemical basis of morphogenesis. *Philos. Trans. R. Soc. Lond. B Biol. Sci.* 237, 37-72. 10.1098/rstb.1952.0012 PMC4360114 25750229

[DEV192914C168] Turner, D. A., Baillie-Johnson, P. and Martinez Arias, A. (2016). Organoids and the genetically encoded self-assembly of embryonic stem cells. *BioEssays* 38, 181-191. 10.1002/bies.201500111 26666846 PMC4737349

[DEV192914C169] Turner, D. A., Girgin, M., Alonso-Crisostomo, L., Trivedi, V., Baillie-Johnson, P., Glodowski, C. R., Hayward, P. C., Collignon, J., Gustavsen, C., Serup, P. et al. (2017). Anteroposterior polarity and elongation in the absence of extra-embryonic tissues and of spatially localised signalling in gastruloids: mammalian embryonic organoids. *Development* 144, 3894-3906. 28951435 10.1242/dev.150391 PMC5702072

[DEV192914C170] van den Brink, S. C., Baillie-Johnson, P., Balayo, T., Hadjantonakis, A.-K., Nowotschin, S., Turner, D. A. and Martinez Arias, A. (2014). Symmetry breaking, germ layer specification and axial organisation in aggregates of mouse embryonic stem cells. *Development* 141, 4231-4242. 10.1242/dev.113001 25371360 PMC4302915

[DEV192914C171] van den Brink, S. C., Alemany, A., van Batenburg, V., Moris, N., Blotenburg, M., Vivié, J., Baillie-Johnson, P., Nichols, J., Sonnen, K. F., Martinez Arias, A. et al. (2020). Single-cell and spatial transcriptomics reveal somitogenesis in gastruloids. *Nature* 582, 405-409. 10.1038/s41586-020-2024-3 32076263

[DEV192914C172] Veenvliet, J. V. and Herrmann, B. G. (2021). Modeling mammalian trunk development in a dish. *Dev. Biol.* 474, 5-15. 10.1016/j.ydbio.2020.12.015 33347872

[DEV192914C173] Veenvliet, J. V., Bolondi, A., Kretzmer, H., Haut, L., Scholze-Wittler, M., Schifferl, D., Koch, F., Guignard, L., Kumar, A. S., Pustet, M. et al. (2020). Mouse embryonic stem cells self-organize into trunk-like structures with neural tube and somites. *Science* 370, eaba4937. 10.1126/science.aba4937 33303587

[DEV192914C174] Vianello, S. and Lutolf, M. P. (2019). Understanding the Mechanobiology of Early Mammalian Development through Bioengineered Models. *Dev. Cell* 48, 751-763. 10.1016/j.devcel.2019.02.024 30913407

[DEV192914C175] Vianello, S. D. and Lutolf, M. (2020). In vitro endoderm emergence and self-organisation in the absence of extraembryonic tissues and embryonic architecture. *bioRxiv*. 10.1101/2020.06.07.138883

[DEV192914C176] Vining, K. H. and Mooney, D. J. (2017). Mechanical forces direct stem cell behaviour in development and regeneration. *Nat. Rev. Mol. Cell Biol.* 18, 728-742. 10.1038/nrm.2017.108 29115301 PMC5803560

[DEV192914C177] Vukicevic, S., Kleinman, H. K., Luyten, F. P., Roberts, A. B., Roche, N. S. and Reddi, A. H. (1992). Identification of multiple active growth factors in basement membrane Matrigel suggests caution in interpretation of cellular activity related to extracellular matrix components. *Exp. Cell Res.* 202, 1-8. 10.1016/0014-4827(92)90397-Q 1511725

[DEV192914C178] Walma, D. A. C. and Yamada, K. M. (2020). The extracellular matrix in development. *Development* 147, dev175596. 10.1242/dev.175596 32467294 PMC7272360

[DEV192914C179] Wollrab, V., Belmonte, J. M., Baldauf, L., Leptin, M., Nédeléc, F. and Koenderink, G. H. (2018). Polarity sorting drives remodeling of actin-myosin networks. *J. Cell Sci.* 132, jcs219717. 10.1242/jcs.219717 30404824

[DEV192914C180] Wolpert, L., Tickle, C. and Martinez Arias, A. (1998). *Principles of Development*. Oxford, UK: Oxford University Press.

[DEV192914C181] Xiong, F., Ma, W., Bénazéraf, B., Mahadevan, L. and Pourquié, O. (2020). Mechanical Coupling Coordinates the Co-elongation of Axial and Paraxial Tissues in Avian Embryos. *Dev. Cell* 55, 354-366.e5. 10.1016/j.devcel.2020.08.007 32918876 PMC7685225

[DEV192914C182] Xu, P. F., Borges, R. M., Fillatre, J., de Oliveira-Melo, M., Cheng, T., Thisse, B. and Thisse, C. (2021). Construction of a mammalian embryo model from stem cells organized by a morphogen signalling centre. *Nat. Commun.* 12, 3277. 10.1038/s41467-021-23653-4 34078907 PMC8172561

[DEV192914C183] Yang, Q., Xue, S.-L., Chan, C. J., Rempfler, M., Vischi, D., Gutierrez, F. M., Hiiragi, T., Hannezo, E. and Liberali, P. (2021). Cell fate coordinates mechano-osmotic forces in intestinal crypt formation. *Nat. Cell Biol.* 23, 733-744. 10.1038/s41556-021-00700-2 34155381 PMC7611267

[DEV192914C184] Zhang, Z., Zwick, S., Loew, E., Grimley, J. S. and Ramanathan, S. (2019). Mouse embryo geometry drives formation of robust signaling gradients through receptor localization. *Nat. Commun.* 10, 4516. 10.1038/s41467-019-12533-7 31586065 PMC6778081

[DEV192914C186] Zheng, Y., Xue, X., Shao, Y., Wang, S., Esfahani, S. N., Li, Z., Muncie, J. M., Lakins, J. N., Weaver, V. M., Gumucio, D. L. et al. (2019). Controlled modelling of human epiblast and amnion development using stem cells. *Nature* 573, 421-425. 10.1038/s41586-019-1535-2 31511693 PMC8106232

